# Interventional hepatic apoC-III knockdown improves atherosclerotic plaque stability and remodeling by triglyceride lowering

**DOI:** 10.1172/jci.insight.158414

**Published:** 2022-07-08

**Authors:** Bastian Ramms, Sohan Patel, Xiaoli Sun, Ariane R. Pessentheiner, G. Michelle Ducasa, Adam E. Mullick, Richard G. Lee, Rosanne M. Crooke, Sotirios Tsimikas, Joseph L. Witztum, Philip L.S.M. Gordts

**Affiliations:** 1 Department of Medicine, University of California, San Diego, La Jolla, California, USA.; 2 Department of Chemistry, Biochemistry I, Bielefeld University, Bielefeld, Germany.; 3 Department of Pharmacology, Mays Cancer Center, Transplant Center, University of Texas Health Science Center at San Antonio, San Antonio, Texas, USA.; 4 Ionis Pharmaceuticals, Carlsbad, California, USA.; 5 Glycobiology Research and Training Center, University of California, San Diego, La Jolla, California, USA.

**Keywords:** Metabolism, Vascular Biology, Atherosclerosis, Cardiovascular disease, Lipoproteins

## Abstract

Apolipoprotein C-III (apoC-III) is a critical regulator of triglyceride metabolism and correlates positively with hypertriglyceridemia and cardiovascular disease (CVD). It remains unclear if therapeutic apoC-III lowering reduces CVD risk and if the CVD correlation depends on the lipid-lowering or antiinflammatory properties. We determined the impact of interventional apoC-III lowering on atherogenesis using an apoC-III antisense oligonucleotide (ASO) in 2 hypertriglyceridemic mouse models where the intervention lowers plasma triglycerides and in a third lipid-refractory model. On a high-cholesterol Western diet apoC-III ASO treatment did not alter atherosclerotic lesion size but did attenuate advanced and unstable plaque development in the triglyceride-responsive mouse models. No lesion size or composition improvement was observed with apoC-III ASO in the lipid-refractory mice. To circumvent confounding effects of continuous high-cholesterol feeding, we tested the impact of interventional apoC-III lowering when switching to a cholesterol-poor diet after 12 weeks of Western diet. In this diet switch regimen, apoC-III ASO treatment significantly reduced plasma triglycerides, atherosclerotic lesion progression, and necrotic core area and increased fibrous cap thickness in lipid-responsive mice. Again, apoC-III ASO treatment did not alter triglyceride levels, lesion development, and lesion composition in lipid-refractory mice after the diet switch. Our findings suggest that interventional apoC-III lowering might be an effective strategy to reduce atherosclerosis lesion size and improve plaque stability when lipid lowering is achieved.

## Graphical abstract

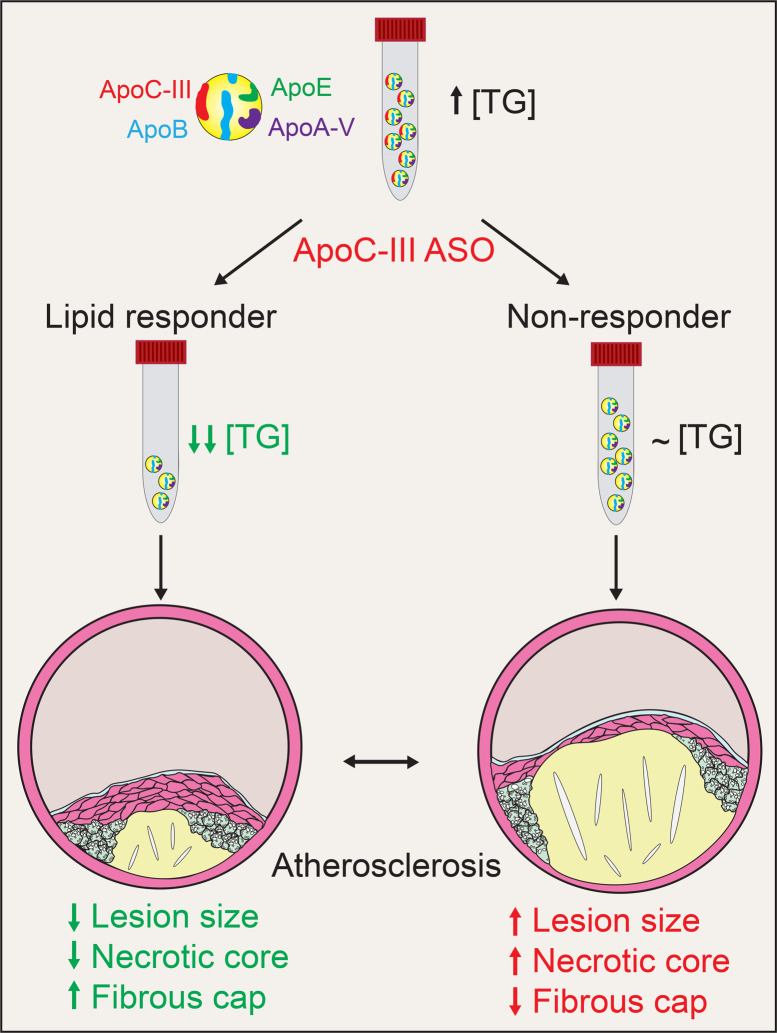

## Introduction

Despite therapeutic improvements, cardiovascular disease (CVD) remains the leading cause of death in the United States and worldwide (1, 2). Current therapeutic interventions are aimed at lowering LDL-cholesterol. However, patients with substantial LDL-cholesterol reduction have persistent residual CVD risk (3). This residual risk has shifted the attention to hypertriglyceridemia (HTG), a condition of elevated plasma triglyceride levels. Genetic studies identified a significant correlation between HTG and CVD risk (3–5). The contribution and mechanisms by which HTG promotes atherosclerosis development, however, remain to be fully elucidated.

Elevated plasma triglycerides, which are transported by triglyceride-rich lipoproteins (TRLs) in the circulation, represent a complex trait in which multiple genetic, metabolic, and environmental factors have been identified, such as lipoprotein lipase (LPL), angiopoietin-like protein 3, proprotein convertase subtilisin/kexin type 9 (PCSK9), and apolipoprotein C-III (apoC-III) (6, 7). Circulating TRLs produced by the intestine, or the liver, undergo LPL-mediated lipolysis, followed by hepatic clearance of TRL remnants mediated by LDL receptor (LDLR), LDLR-related protein 1 (LRP1), and syndecan 1 (SDC1) (8). ApoC-III, an 8.8 kDa glycoprotein mainly produced in the liver and associated with TRLs, LDL, and HDL particles, is a well-established regulating factor of triglyceride metabolism (9, 10). In vivo, apoC-III inhibits hepatic clearance of TRLs through the LDLR/LRP1 axis (11, 12). In contrast, hepatic SDC1 clears apoC-III–enriched TRLs, and consequently, inactivation of liver SDC1 results in elevated plasma apoC-III levels (11, 12). ApoC-III also inhibits LPL activity and may promote VLDL production (13–16). ApoC-III requires the TRL receptor ligand apolipoprotein E (apoE) to inhibit hepatic TRL clearance. In the absence of *Apoe* expression, apoC-III lowering improves LPL activity in adipose tissue (17).

Besides its role in HTG, apoC-III has been suggested to affect other CVD-related risk factors (6). In fact, apoC-III–induced signaling through NF-κB stimulates vascular cell adhesion molecule 1 expression and subsequent endothelium activation (18). Furthermore, apoC-III was shown to promote inflammation through upregulation of proinflammatory cytokines, such as IL-6 and TNF-α, resulting in endothelial cell dysfunction (19–21). Recent work reported that apoC-III activates an alternative NLR family pyrin domain containing 3 inflammasome pathway in monocytes through TLR2 and TLR4 dimerization, stimulating sterile inflammation (22). In this fashion, apoC-III can potentially promote activation and accumulation of monocytes and macrophages and enhance the adhesion of monocytes to endothelial cells independent of the impact on plasma TRL levels (20, 21, 23, 24).

The importance of apoC-III in CVD became evident when inactivating mutations affecting its expression in humans correlated with lower plasma triglycerides and protection against CVD (25–27). These observations spurred the development of therapeutics targeting apoC-III plasma levels in humans. This includes a human *Apoc3* antisense oligonucleotide (ASO) that dramatically lowers plasma triglycerides in patients with HTG and familial chylomicronemia syndrome (10, 12, 28, 29). Although the impact of therapeutic apoC-III lowering on HTG is well established, the benefit of interventional apoC-III targeting to reduce atherosclerosis development and promote plaque progression and regression remains unclear. Furthermore, the plethora of mechanisms by which apoC-III can contribute to atherosclerosis development raises the question as to which of these pathways is predominantly responsible for the apoC-III–associated CVD risk.

**Figure 1:**
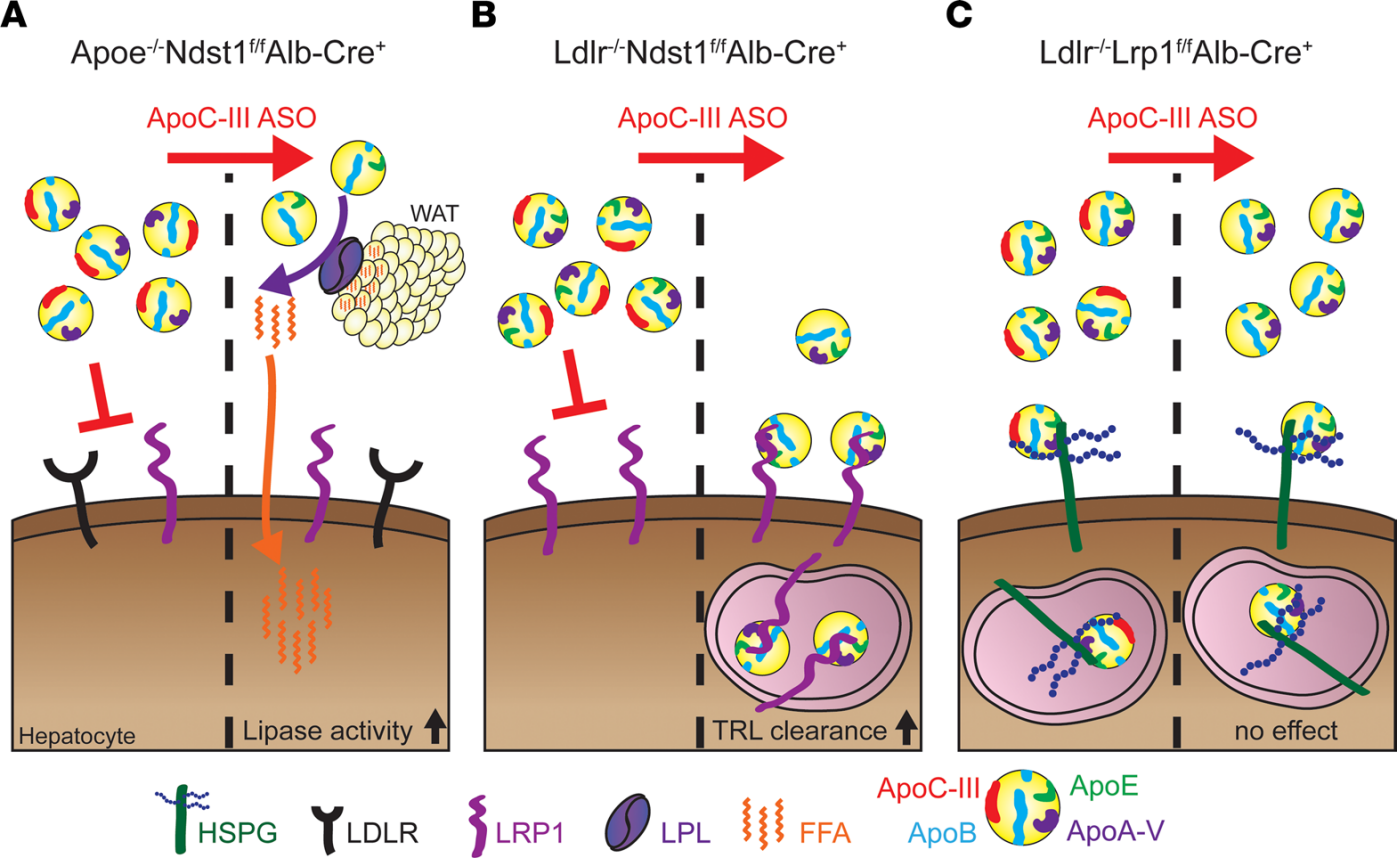
Effect of liver apoC-III targeting on lipid lowering. (**A**) In the absence of apoE-mediated triglyceride-rich lipoprotein (TRL) clearance (*Apoe*^–/–^ *Ndst1*^fl/fl^ *Alb-Cre*^+^), apoC-III ASO lowers plasma triglyceride levels by promoting LPL activity in white adipose tissue (WAT), resulting in increased uptake of free fatty acids (FFAs) into the WAT and liver (11, 12). *Ndst1*, N-deacetylase and N-sulfotransferase 1. (**B**) ApoC-III ASO administration results in improved hepatic TRL clearance mediated by LDLR-related protein 1 (LRP1) in the absence of LDLR and heparan sulfate proteoglycan (HSPG) receptor (*Ldlr*^–/–^ *Ndst1*^fl/fl^ *Alb-Cre*^+^) (11, 12). (**C**) In contrast, targeting apoC-III with ASOs has no impact on TRL clearance when both LDLR and LRP1 are deficient (11, 17).

In the current study, we targeted hepatic *Apoc3* expression with liver-specific ASOs and analyzed its effect on atherosclerosis development and progression dependent or independent of its triglyceride-lowering properties using mouse models (Figure 1, A–C) (11, 17). We find that despite substantial triglyceride lowering in mice, apoC-III inhibition did not attenuate atherosclerotic lesion size development upon feeding with a high-cholesterol diet. However, analysis of the plaque composition revealed that apoC-III lowering significantly reduced necrotic core area and resulted in plaque remodeling, including fibrous cap thickening when apoC-III also lowered plasma TRL levels. Furthermore, in a low-cholesterol diet intervention model, we found that the progression of atherosclerosis could be halted by apoC-III inhibition, which was associated with improved TRL clearance. Overall, the data suggest that apoC-III reduced necrotic core area by enhanced TRL clearance. The more stable lesion phenotype will reduce the incidence of plaque rupture, which can contribute to the positive correlation between apoC-III levels and acute coronary disease events in humans.

## Results

### Interventional apoC-III lowering does not improve atherogenesis in hyperlipidemic mice fed a low-fat diet.

**Figure 2:**
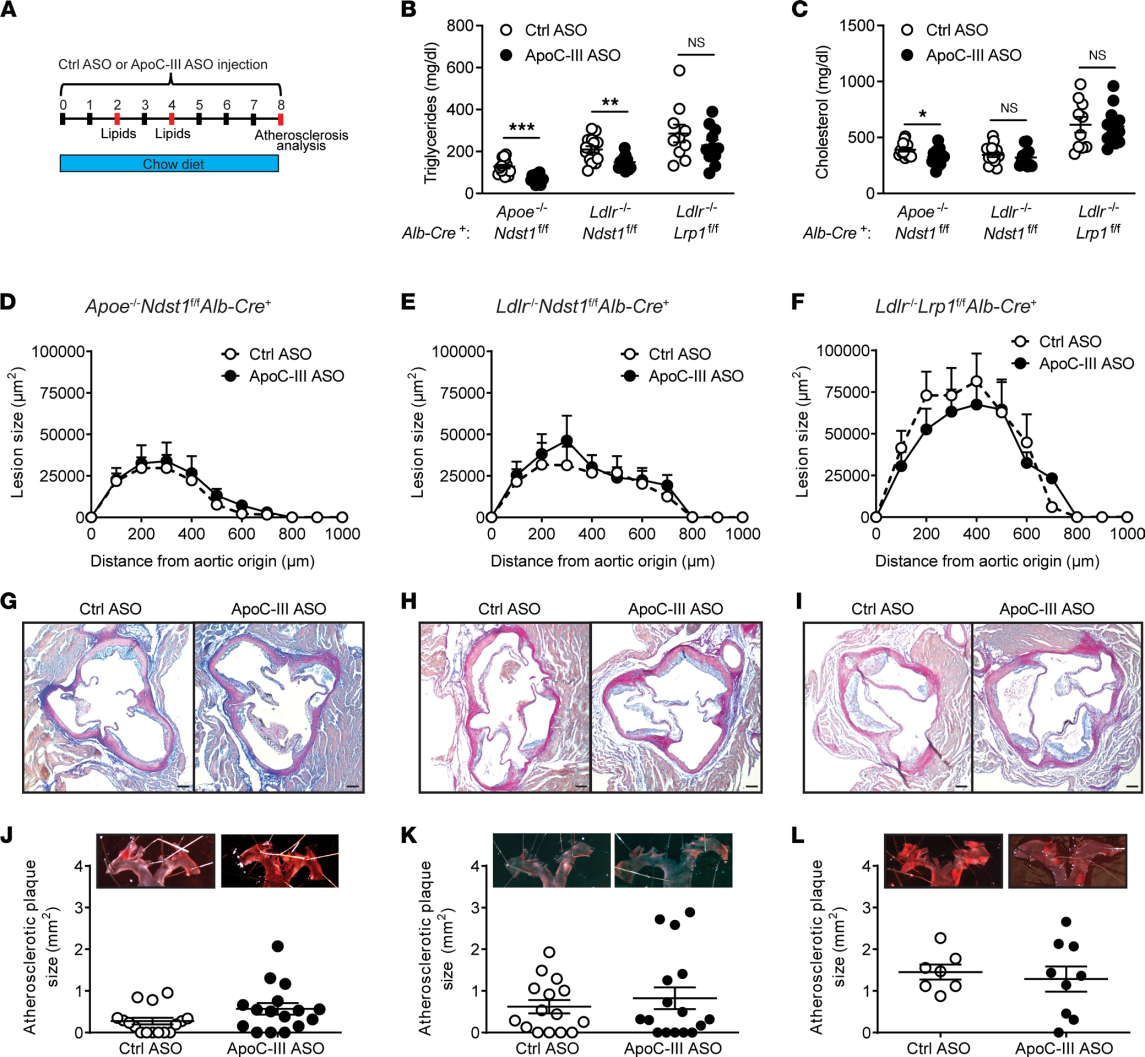
ApoC-III targeting does not lower atherosclerotic lesion size and area in chow-fed mice. (**A**) Twelve-week-old *Apoe*^–/–^ *Ndst1*^fl/fl^ *Alb*-*Cre*^+^, *Ldlr*^–/–^ *Ndst1*^fl/fl^ *Alb*-*Cre*^+^, and *Ldlr*^–/–^ *Lrp1*^fl/fl^ *Alb*-*Cre*^+^ chow-fed mice were intraperitoneally injected once weekly with control ASO or apoC-III ASO (50 mg/kg body weight). Lipids were monitored at the indicated time points and atherosclerosis was assessed after 8 weeks. (**B**) Fasting plasma triglyceride levels after 8 weeks of apoC-III ASO treatment in *Apoe*^–/–^ *Ndst1*^fl/fl^ *Alb*-*Cre*^+^ (*n* = 18–19/group), *Ldlr*^–/–^ *Ndst1*^fl/fl^ *Alb*-*Cre*^+^ (*n* = 13–16/group), and *Ldlr*^–/–^ *Lrp1*^fl/fl^ *Alb*-*Cre*^+^ mice (*n* = 10–12/group). (**C**) Fasting plasma cholesterol levels. (**D**–**F**) Assessment of lesion formation with modified Verhoeff-van Gieson stain in the aortic root after 8 weeks of control ASO or apoC-III ASO in (**D**) *Apoe*^–/–^ *Ndst1*^fl/fl^ *Alb*-*Cre*^+^ (*n* = 16/group), (**E**) *Ldlr*^–/–^ *Ndst1*^fl/fl^ *Alb*-*Cre*^+^ (*n* = 15–16/group), and (**F**) *Ldlr*^–/–^ *Lrp1*^fl/fl^ *Alb*-*Cre*^+^ (*n* = 7–9/group) mice. (**G**–**I**) Representative images of aortic root sections in all 3 models. (**J**–**L**) En face analysis of the aortas for (**J**) *Apoe*^–/–^ *Ndst1*^fl/fl^ *Alb*-*Cre*^+^, (**K**) *Ldlr*^–/–^ *Ndst1*^fl/fl^ *Alb*-*Cre*^+^, and (**L**) *Ldlr*^–/–^ *Lrp1*^fl/fl^ *Alb*-*Cre*^+^ mice as analyzed by Sudan IV stain. Data presented as mean ± SEM. Statistical differences between 2 groups were calculated using an unpaired 2-tailed Student’s *t* test. Results on lesion size over distance were analyzed using a 2-way ANOVA followed by Bonferroni’s post hoc test. *P < 0.05, **P < 0.01, ***P < 0.001.

To determine if apoC-III–knockdown strategies can improve atherosclerosis, we administered an ASO against *Apoc3* (50 mg/kg/w) to mouse models that differ in their metabolic response to apoC-III inhibition (Figure 1, A–C). First, apoC-III ASO lowered plasma triglyceride levels in *Apoe*^–/–^ *Ndst1*^fl/fl^ *Alb-Cre*^+^ mice by promoting tissue LPL activity, independent of its effects on hepatic TRL clearance (Figure 1A) (17). Second, in *Ldlr*^–/–^ *Ndst1*^fl/fl^ *Alb-Cre*^+^ mice, apoC-III inhibition lowered triglycerides by reducing TRL lipoprotein particle numbers, improving hepatic TRL clearance via LRP1 (Figure 1B) (11). In these mice, TRL size was not altered, and no improvement of LPL activity was observed upon apoC-III ASO treatment (11). Third, in contrast, upon apoC-III ASO administration, plasma lipid levels were unaffected in *Ldlr*^–/–^ *Lrp1*^fl/fl^ *Alb-Cre*^+^ mice, which lack both hepatic LDLR and LRP1 receptors (Figure 1C) (11). The first 2 models allowed us to study the impact of apoC-III on atherogenesis dependent on a reduction in triglyceride levels, while the third model evaluated a possible role of the proinflammatory properties of apoC-III in atherosclerosis development, independent of changes in plasma triglycerides. To determine the impact of apoC-III lowering on atherogenesis, we assessed atherosclerosis development after 8 weeks of apoC-III ASO treatment. Initially, we analyzed the impact on atherosclerosis development in 12-week-old mice receiving a cholesterol-poor standard diet (Figure 2A). This was done to prevent the substantial hypercholesterolemia induced by Western diet feeding from masking the impact of apoC-III lowering on atherogenesis.

On a standard chow diet, administration of apoC-III ASO downregulated hepatic *Apoc3* gene expression compared with control ASO by 87.5% ± 2.0% (mean ± SEM, *P* < 0.0001) in *Apoe*^–/–^ *Ndst1*^fl/fl^ *Alb-Cre*^+^ mice, by 93.3% ± 1.6% (*P* = 0.0004) in *Ldlr*^–/–^ *Ndst1*^fl/fl^ *Alb-Cre*^+^ mice, and by 83.9% ± 3.2% (*P* = 0.0001) in *Ldlr*^–/–^ *Lrp1*^fl/fl^ *Alb-Cre*^+^ mice, respectively (Supplemental Figure 1A; supplemental material available online with this article; https://doi.org/10.1172/jci.insight.158414DS1), consistent with previous studies (11, 17). Knockdown of apoC-III was confirmed by analyzing its presence on TRLs by Western blotting (Supplemental Figure 1B). The reduction in *Apoc3* significantly decreased plasma triglyceride levels by 45.3% ± 3.7% (*P* < 0.0001) in *Apoe*^–/–^ *Ndst1*^fl/fl^ *Alb-Cre*^+^ mice and by 29.1% ± 4.0% (*P* = 0.0022) in *Ldlr*^–/–^ *Ndst1*^fl/fl^ *Alb-Cre*^+^ mice, respectively, compared with control ASO after 8 weeks of ASO administration (Figure 2B and Supplemental Figure 1C). The reduction in plasma triglyceride levels was due to reduced triglycerides in the chylomicron remnant and TRL fraction as determined by fast performance liquid chromatography (FPLC) (11, 17). Similar results were obtained after 4 weeks of ASO treatment (Supplemental Figure 1, D and E). In contrast, in the absence of LDLR and LRP1, apoC-III lowering did not significantly alter plasma triglyceride levels (–19.9% ± 9.2%, *P* = 0.26) (Figure 2B and Supplemental Figure 1C). Cholesterol was significantly reduced by 14.5% ± 5.2% (*P* = 0.029) in *Apoe*^–/–^ *Ndst1*^fl/fl^ *Alb-Cre*^+^ mice but unaffected in the other 2 models (Figure 2C and Supplemental Figure 1F). No differences in body weight were observed between control ASO and apoC-III ASO in all studied models (Supplemental Figure 1, G–I).

To determine the impact of apoC-III lowering on early stages of atherogenesis, we assessed atherosclerosis development after 8 weeks of ASO treatment by analyzing the aortic root and via en face analysis of the aorta. In all 3 models, even on control chow diet, the mice had cholesterol levels of 400–600 mg/dL (Figure 2C), and we observed development of atherosclerotic lesions around the aortic root and the aortic arch (Figure 2, D–L). Interestingly, apoC-III ASO–mediated triglyceride lowering did not improve early-onset atherogenesis in *Apoe*^–/–^ *Ndst1*^fl/fl^ *Alb-Cre*^+^ (Figure 2, D, G, and J) and *Ldlr*^–/–^ *Ndst1*^fl/fl^ *Alb-Cre*^+^ mice (Figure 2, E, H, and K). Similar results were obtained from mice lacking LDLR and LRP1, in which apoC-III lowering did not affect plasma triglycerides (Figure 2, F, I, and L). In the double LDLR and LRP1 knockout model, absolute atherosclerotic lesion size was increased compared with lesions in *Apoe*^–/–^ *Ndst1*^fl/fl^ *Alb-Cre*^+^ and *Ldlr*^–/–^ *Ndst1*^fl/fl^ *Alb-Cre*^+^ mice (Figure 2, D and E), which correlated with their elevated plasma cholesterol levels and triglyceride levels.

### ApoC-III ASO administration does not attenuate atherosclerotic lesion size in Western diet–fed hyperlipidemic mice.

**Figure 3:**
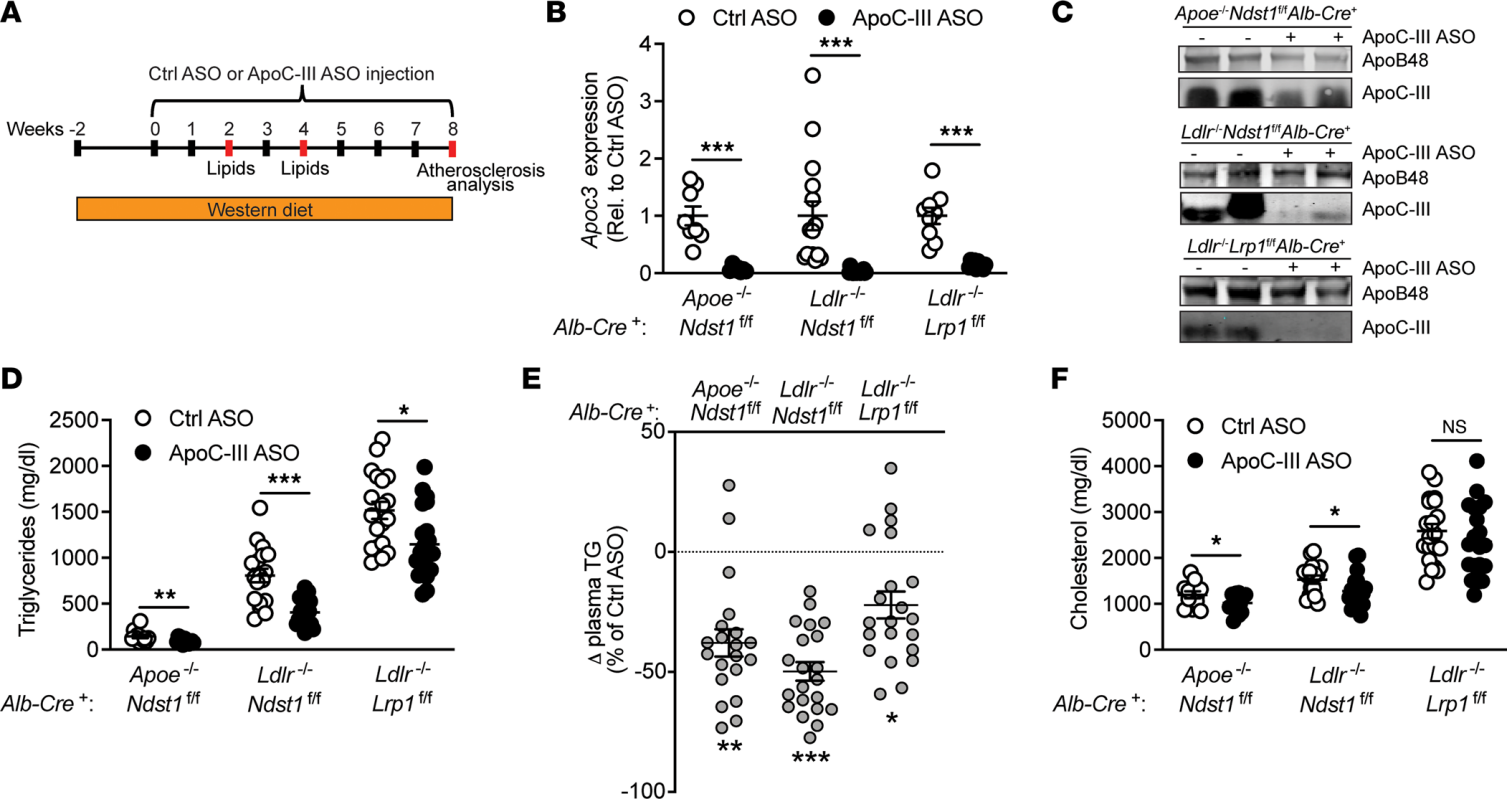
Impact of the apoC-III ASO on lipid lowering upon Western diet feeding. (**A**) Twelve-week-old mice were fed a Western diet for 10 weeks with either control ASO or apoC-III ASO (50 mg/kg body weight) treatment beginning after 2 weeks of Western diet feeding. ASOs were injected intraperitoneally once weekly to *Apoe*^–/–^ *Ndst1*^fl/fl^ *Alb*-*Cre*^+^ (*n* = 17–20/group), *Ldlr*^–/–^ *Ndst1*^fl/fl^ *Alb*-*Cre*^+^ (*n* = 18–22/group), and *Ldlr*^–/–^ *Lrp1*^fl/fl^ *Alb*-*Cre*^+^ mice (*n* = 19–22/group). Fasting lipid levels were analyzed after 2, 4, and 8 weeks of treatment (red). (**B**) Hepatic *Apoc3* gene expression relative to control ASO. (**C**) Detection of apolipoproteins (apoB, apoC-III) in pooled plasma samples (2 μL, *n* = 3/pool) by Western blotting. (**D**) Fasting plasma triglyceride levels after 8 weeks of control ASO or apoC-III ASO. (**E**) Relative change in plasma triglyceride levels compared to control ASO. (**F**) Fasting plasma cholesterol levels after 8 weeks of control ASO or apoC-III ASO. Data presented as mean ± SEM. Statistical differences between 2 groups were calculated using an unpaired 2-tailed Student’s *t* test. *P < 0.05, **P < 0.01, ***P < 0.001.

Chow-fed mice studied above developed early atherosclerotic lesions or fatty streaks, hallmarked by accumulation of foam cells in the subendothelial space. We concluded that the observed lesions might be too early to detect differences upon short-term apoC-III ASO administration. Hence, we fed mice a Western diet for 10 weeks to induce more advanced atherosclerotic lesions (Figure 3A). Two weeks after starting the Western diet, mice received control or apoC-III ASO treatments for 8 weeks. Starting the Western diet feeding 2 weeks before apoC-III ASO injections was based on the idea of raising plasma lipid levels and starting atherogenesis before therapeutic intervention, analogous to a clinical setting, in which a patient receives drug treatment after disease onset or identification of the presence of significant risk factors. *Apoc3* knockdown was confirmed by quantitative PCR (Figure 3B) and Western blotting (Figure 3C). The downregulation of *Apoc3* resulted in a reduction in plasma triglyceride levels by 37.9% ± 5.7% (*P* = 0.0068) in *Apoe*^–/–^ *Ndst1*^fl/fl^ *Alb-Cre*^+^ mice and by 49.8% ± 3.9% (*P* < 0.0001) in *Ldlr*^–/–^ *Ndst1*^fl/fl^ *Alb-Cre*^+^ mice, respectively (Figure 3, D and E), which is explained by a reduction of triglycerides in the chylomicron remnant and VLDL fraction (11, 17). In contrast to chow-fed mice, plasma triglycerides were also decreased in *Ldlr*^–/–^ *Lrp1*^fl/fl^ *Alb-Cre*^+^ mice by 22.1% ± 5.46% (*P* = 0.010) (Figure 3, D and E) upon apoC-III ASO administration despite absolute triglyceride levels over 2000 mg/dL. However, no changes in plasma triglycerides were observed after 4 weeks or 6 weeks as described previously (Supplemental Figure 2, A and B) (11). Compared with chow diet, on the Western diet, plasma cholesterol levels were significantly increased in all 3 mouse models. However, apoC-III targeting resulted in minor plasma cholesterol lowering in *Apoe*^–/–^ *Ndst1*^fl/fl^ *Alb-Cre*^+^ and *Ldlr*^–/–^ *Ndst1*^fl/fl^ *Alb-Cre*^+^ mice, respectively, but not in *Ldlr*^–/–^ *Lrp1*^fl/fl^ *Alb-Cre*^+^ mice (Figure 3F and Supplemental Figure 2C). FPLC profiling of lipoprotein subclasses revealed a cholesterol reduction in the chylomicron remnant and VLDL fraction for *Apoe*^–/–^ *Ndst1*^fl/fl^ *Alb-Cre*^+^ mice (17) and both the chylomicron remnant/VLDL and IDL/LDL fractions for *Ldlr*^–/–^ *Ndst1*^fl/fl^ *Alb-Cre*^+^ mice, whereas no differences were measured in *Ldlr*^–/–^ *Lrp1*^fl/fl^ *Alb-Cre*^+^ mice (Supplemental Figure 2, D and E). ApoC-III ASO treatment did not affect the body weight in any of the 3 models compared to control ASO (Supplemental Figure 2, F–H).

**Figure 4:**
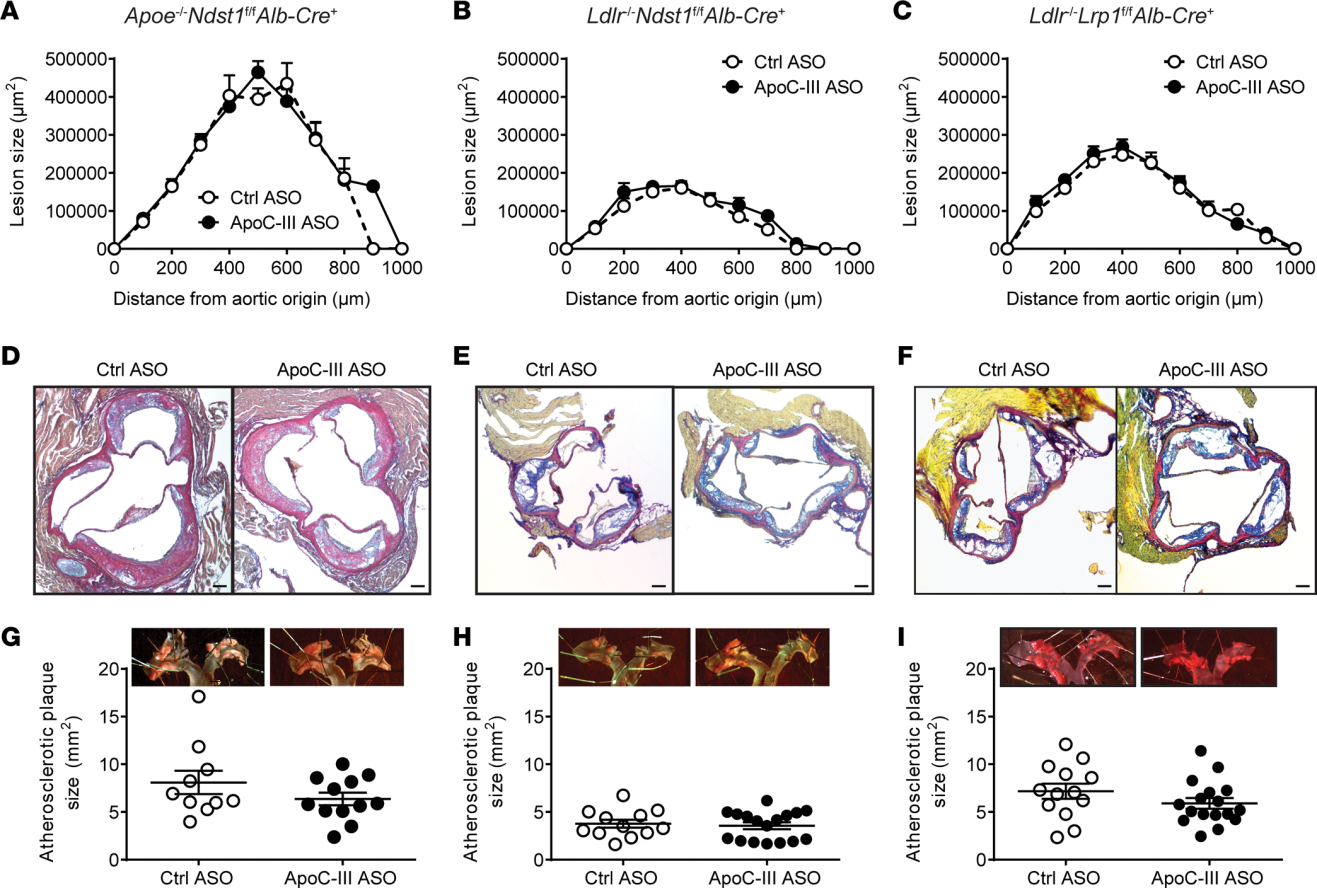
Impact of therapeutic apoC-III lowering on atherosclerosis development in mice fed a Western diet. (**A**–**C**) Atherosclerotic lesion size in (**A**) *Apoe*^–/–^ *Ndst1*^fl/fl^ *Alb*-*Cre*^+^ (*n* = 10–12/group), (**B**) *Ldlr*^–/–^ *Ndst1*^fl/fl^ *Alb*-*Cre*^+^ (*n* = 12–17/group), and (**C**) *Ldlr*^–/–^ *Lrp1*^fl/fl^ *Alb*-*Cre*^+^ mice (*n* = 13–17/group) using modified Verhoeff-van Gieson stain. (**D**–**F**) Examples of aortic root lesion formation in the respective mouse model. (**G**–**I**) Plaque area was determined by en face analysis of the aorta using Sudan IV stain in (**G**) *Apoe*^–/–^ *Ndst1*^fl/fl^ *Alb*-*Cre*^+^, (**H**) *Ldlr*^–/–^ *Ndst1*^fl/fl^ *Alb*-*Cre*^+^, and (**I**) *Ldlr*^–/–^ *Lrp1*^fl/fl^ *Alb*-*Cre*^+^ mice. Representative images of the aortic arch of the corresponding mice treated with either control ASO or apoC-III ASO. Data presented as mean ± SEM. Statistical differences between 2 groups were calculated using an unpaired 2-tailed Student’s *t* test. Results on lesion size over distance were analyzed using a 2-way ANOVA followed by Bonferroni’s post hoc test.

Western diet feeding induced more advanced atherosclerotic lesion development in all 3 studied models (Figure 4, A–I). However, no differences in lesion size were measured in mice treated with apoC-III ASO compared to control ASO. *Apoe*^–/–^ *Ndst1*^fl/fl^ *Alb-Cre*^+^ mice showed the largest lesion development (control ASO: 221.3 mm^2^, apoC-III ASO: 239.6 mm^2^) compared with *Ldlr*^–/–^ *Ndst1*^fl/fl^ *Alb-Cre*^+^ (control ASO: 73.8 mm^2^; apoC-III ASO: 88.0 mm^2^) and *Ldlr*^–/–^ *Lrp1*^fl/fl^ *Alb-Cre*^+^ mice (control ASO: 135.6 mm^2^; apoC-III ASO: 143.3 mm^2^) (Figure 4, A–F). ApoC-III ASO did not alter aortic plaque area compared to control ASO–treated groups as determined by en face analysis (Figure 4, G–I). ApoC-III ASO had no impact on aorta vessel properties as determined by measuring the total vessel area (Supplemental Figure 3, A–D), the lumen area (Supplemental Figure 3E), and the thickness of the aortic vessel wall (Supplemental Figure 3F). Taken together, our results show that short-term apoC-III targeting with ASOs improved neither atherosclerotic lesion size nor volume.

### ApoC-III ASO–mediated accelerated hepatic TRL clearance reduces atherosclerotic necrotic core area.

**Figure 5:**
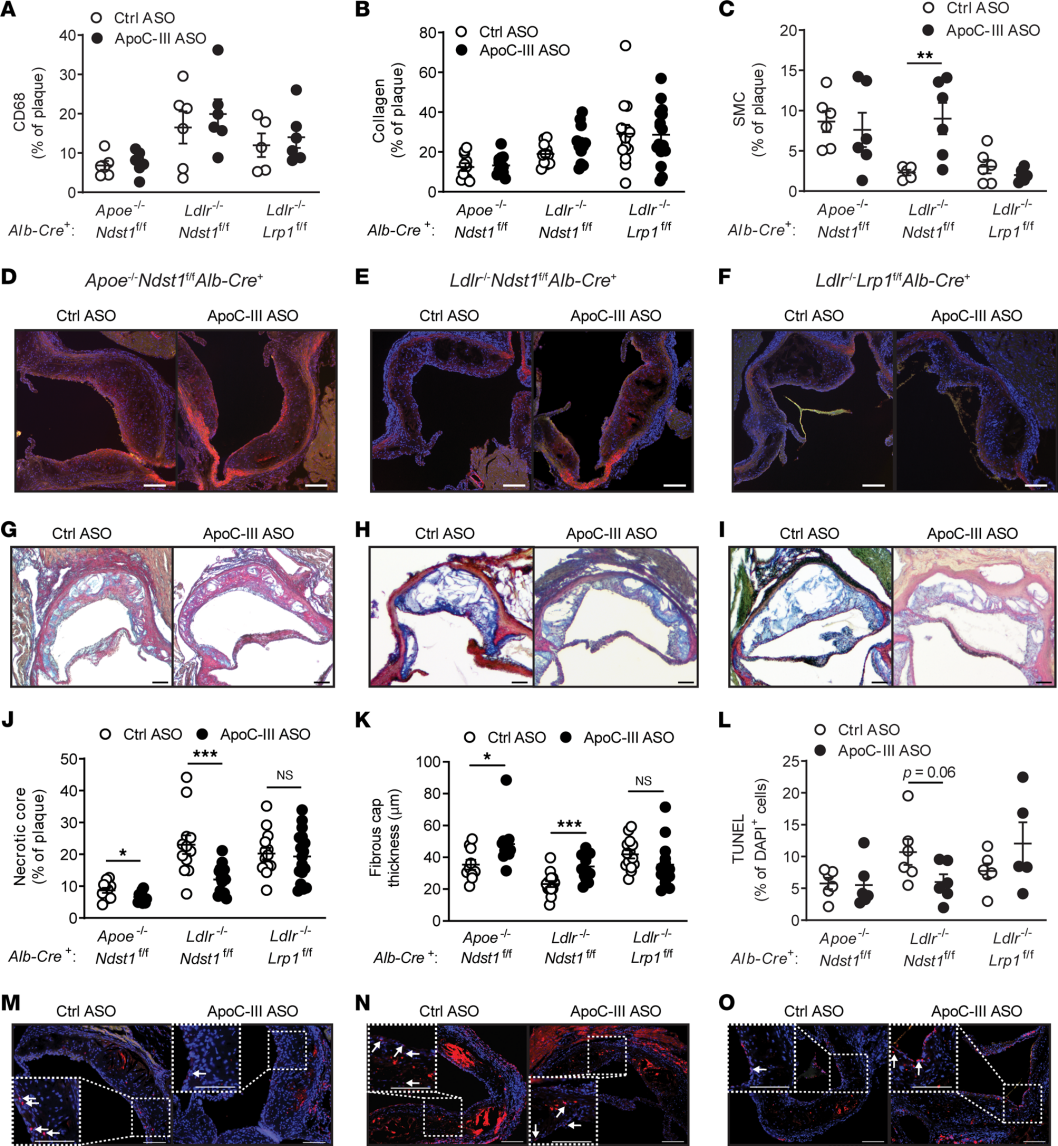
ApoC-III ASO–mediated lowering of triglycerides increases markers of plaque stability. *Apoe*^–/–^*Ndst1*^fl/fl^*Alb*-*Cre*^+^, *Ldlr*^–/–^*Ndst1*^fl/fl^*Alb*-*Cre*^+^, and *Ldlr*^–/–^*Lrp1*^fl/fl^*Alb*-*Cre*^+^ mice fed a Western diet were administered with control ASO or apoC-III ASO for 8 weeks, and aortic root cross-sectional lesions (300 μm) were stained with (**A**) anti-CD68 antibody (*n* = 6/group), (**B**) modified Verhoeff-van Gieson (*n* = 11–16/group), and (**C**) anti-SMC antibody (*n* = 4–6/group). (**D**–**F**) Representative images of SMC detection in (**D**) *Apoe*^–/–^ *Ndst1*^fl/fl^ *Alb*-*Cre*^+^, (**E**) *Ldlr*^–/–^ *Ndst1*^fl/fl^ *Alb*-*Cre*^+^, and (**F**) *Ldlr*^–/–^ *Lrp1*^fl/fl^ *Alb*-*Cre*^+^ mice. (**G**–**I**) Examples of necrotic core areas. (**J**) Quantification of necrotic core area in lesions quantified as a percentage of the plaque size (*n* = 11–17/group). (**K**) Quantification of the fibrous cap (*n* = 10–17/group). (**L**) Apoptotic cells were quantified using a TUNEL stain and DAPI (*n* = 5–6/group). Representative images are shown (**M**) for *Apoe*^–/–^ *Ndst1*^fl/fl^ *Alb*-*Cre*^+^, (**N**) *Ldlr*^–/–^ *Ndst1*^fl/fl^ *Alb*-*Cre*^+^, and (**O**) *Ldlr*^–/–^ *Lrp1*^fl/fl^ *Alb*-*Cre*^+^ mice. Arrows indicate TUNEL-positive cells. Scale bar equals 100 μm. Data presented as mean ± SEM. Statistical differences between 2 groups were calculated using an unpaired 2-tailed Student’s *t* test. *P < 0.05, **P < 0.01, ***P < 0.001.

Next, we wanted to determine if interventional apoC-III lowering affected atherosclerotic lesion composition and stability by analyzing aortic root cross sections from the Western diet–fed mice. ApoC-III ASO did not alter macrophage lesion content as determined by CD68 staining (Figure 5A and Supplemental Figure 4, A–D). Migration and proliferation of smooth muscle cells (SMCs) into the tunica intima of the lesion play an important role in stabilizing the plaque by producing extracellular matrix proteins, such as collagen, and through formation of a protective cap (30, 31). Although collagen content of the lesions was unaffected by targeting apoC-III (Figure 5B), we found that in *Ldlr*^–/–^ *Ndst1*^fl/fl^ *Alb-Cre*^+^ mice apoC-III ASO–mediated triglyceride lowering correlated with a significant 3.9-fold increase in SMC content (*P* = 0.008) within the plaque compared with control ASO treatment (Figure 5, C–F, and Supplemental Figure 4E). No SMC content changes were observed between apoC-III ASO and control ASO treatment in *Apoe*^–/–^ *Ndst1*^fl/fl^ *Alb-Cre*^+^ and *Ldlr*^–/–^ *Lrp1*^fl/fl^ *Alb-Cre*^+^ mice (Figure 5, C–F).

Progression of atherosclerosis is characterized by increased macrophage apoptosis and defective efferocytosis resulting in detrimental expansion of the necrotic core, causing plaque disruption and subsequent luminal thrombosis (32, 33). Analysis of the necrotic core area (Figure 5, G–J) in *Apoe*^–/–^ *Ndst1*^fl/fl^ *Alb-Cre*^+^ mice revealed that apoC-III ASO reduced necrotic core area significantly by 24.3% ± 28.0% (*P* = 0.036, Figure 5, G and J). Furthermore, in *Ldlr*^–/–^ *Ndst1*^fl/fl^ *Alb-Cre*^+^ mice, apoC-III ASO–mediated triglyceride lowering was associated with an even more pronounced 47.5% ± 5.2% reduction in necrotic core area (*P* = 0.0008, Figure 5, H and J). It is worth noting that these differences are independent of the lesion size (Figure 4, A–C). Furthermore, apoC-III ASO treatment resulted in a significant 37% and 47% thicker fibrous cap surrounding the necrotic cores in *Apoe*^–/–^ *Ndst1*^fl/fl^ *Alb-Cre*^+^ and *Ldlr*^–/–^ *Ndst1*^fl/fl^ *Alb-Cre*^+^ mice, respectively (Figure 5K). In contrast, targeting apoC-III had no effect on necrotic core area or fibrous cap thickness compared to control ASO in *Ldlr*^–/–^ *Lrp1*^fl/fl^ *Alb-Cre*^+^ mice (Figure 5, I–K). Recently, oxidized phospholipids (oxPLs) were identified as a driver of atherogenesis (34). Using a specific antibody (E06) to detect hydrophilic phosphocholine groups in oxPLs (34), we analyzed the oxPL content in atherosclerotic lesions in the 3 used mouse models (Supplemental Figure 4, F–I). However, apoC-III did not affect oxPL content in atherosclerotic lesions. Thus, the reduction in necrotic core area is not reflected by changes in oxPL. Apoptotic cell content within atherosclerotic lesions were analyzed using terminal deoxynucleotidyl transferase–mediated dUTP nick end labeling (TUNEL). *Ldlr*^–/–^ *Ndst1*^fl/fl^ *Alb-Cre*^+^ mice on apoC-III ASO showed a decrease in TUNEL-positive cells by 44.0% ± 11.4% (*P* = 0.06), corresponding to smaller necrotic cores. In contrast, apoC-III ASO did not affect apoptosis in *Apoe*^–/–^ *Ndst1*^fl/fl^ *Alb-Cre*^+^ (*P* = 0.92) and *Ldlr*^–/–^ *Lrp1*^fl/fl^ *Alb-Cre*^+^ mice (*P* = 0.10, Figure 5, L–O, and Supplemental Figure 4J). Together, our results suggest that apoC-III ASO–mediated TRL clearance improves markers of atherosclerotic plaque stability as it is associated with increased SMC content and a reduced necrotic core area in *Ldlr*^–/–^ *Ndst1*^fl/fl^ *Alb-Cre*^+^ mice.

### ApoC-III ASO improves insulin sensitivity in Ldlr^–/–^ Ndst1^fl/fl^ Alb-Cre^+^ mice.

**Figure 6:**
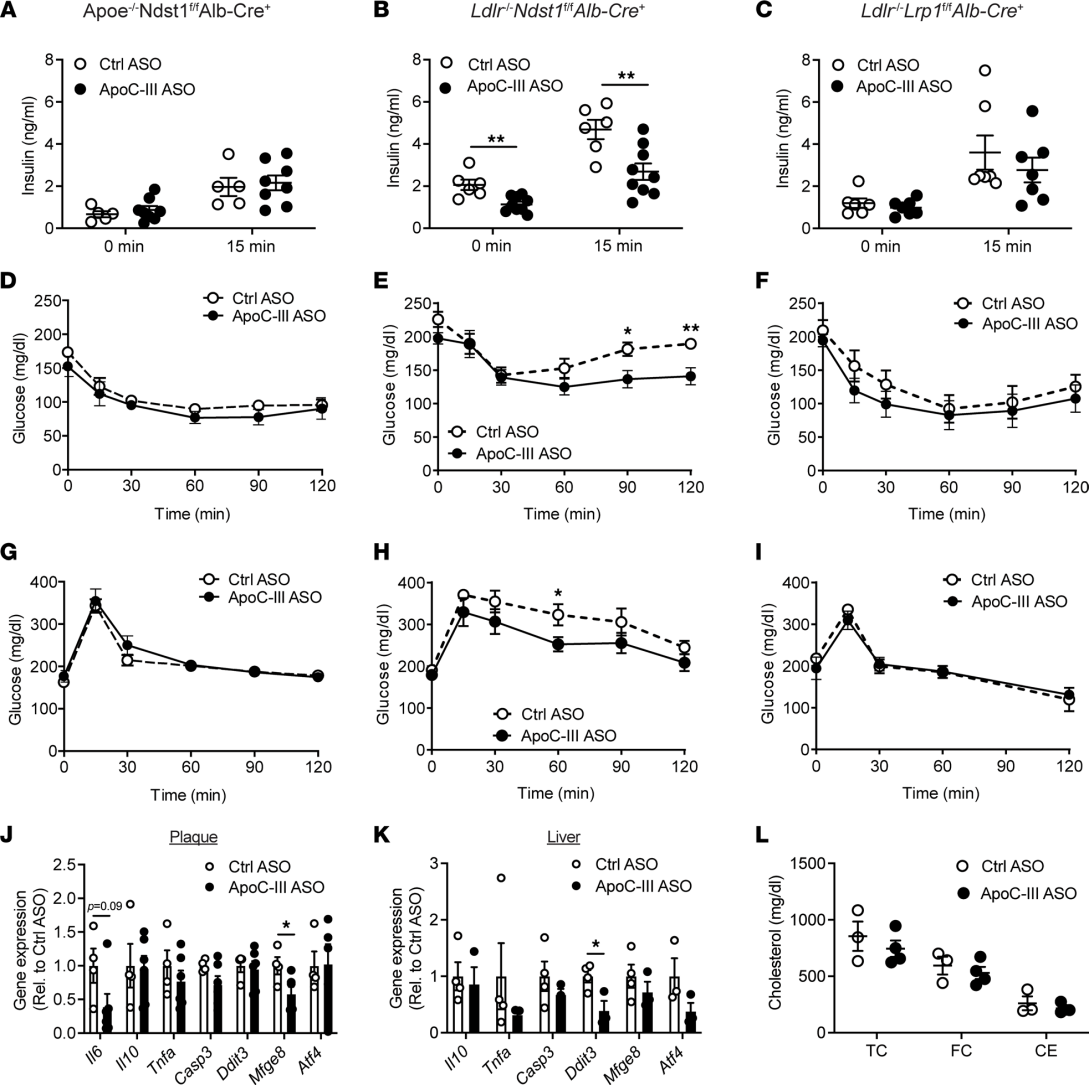
ApoC-III ASO improves insulin sensitivity in *Ldlr*^–/–^ *Ndst1*^fl/fl^ *Alb-Cre*^+^ mice. Measurements of glucose homeostasis were performed after 6–8 weeks of control ASO or apoC-III ASO treatment in mice fed a Western diet. (**A**–**C**) Plasma insulin concentrations were determined via ELISA in fasted (**A**) *Apoe*^–/–^ *Ndst1*^fl/fl^ *Alb*-*Cre*^+^, (**B**) *Ldlr*^–/–^ *Ndst1*^fl/fl^ *Alb*-*Cre*^+^, and (**C**) *Ldlr*^–/–^ *Lrp1*^fl/fl^ *Alb*-*Cre*^+^ mice and 15 minutes after glucose gavage (2 mg/g body weight, *n* = 6–9/group). (**D**–**F**) Insulin tolerance test (*n* = 5–6/group) and (**G**–**I**) glucose tolerance test (*n* = 4–6/group) were performed by intraperitoneal injection of 0.6 U/kg body weight insulin or oral gavage with 2 mg/g body weight glucose, and blood glucose levels were measured at 0, 15, 30, 60, 90, and 120 minutes. (**J** and **K**) Gene expression of markers of inflammation (*Il6*, *Il10*, *Tnfa*), apoptosis (caspase-3, *Casp3*), and ER stress (*Ddit3*, *Atf4*) were analyzed in (**J**) atherosclerotic plaques (*n* = 3–4/group) and (**K**) liver (*n* = 3–4/group) of *Ldlr*^–/–^ *Ndst1*^fl/fl^ *Alb*-*Cre*^+^ mice. Values are expressed relative to control ASO. *Mfge8*, milk fat globule EGF and factor V/VIII domain containing. (**L**) Peritoneal macrophages were isolated from *Ldlr*^–/–^ *Ndst1*^fl/fl^ *Alb*-*Cre*^+^ mice, and total cholesterol (TC), free cholesterol (FC), and cholesteryl ester (CE) were measured (*n* = 3–4/group). Data presented as mean ± SEM. Response to metabolic challenges over time was calculated using a 2-way ANOVA with Fisher’s least significant difference post hoc analysis. Statistical differences between 2 groups were calculated using an unpaired 2-tailed Student’s *t* test. *P < 0.05, **P < 0.01.

Insulin resistance, a hallmark of diet-induced type 2 diabetes, induces cellular ER stress, a driver of macrophage apoptosis and necrotic core formation (32). Previous studies have reported a correlation between increased apoC-III levels and insulin resistance and CVD risk in type 1 diabetes (35, 36). Hence, we evaluated whether the impact of apoC-III targeting on glucose homeostasis depended on its lipid-lowering properties (Figure 6, A–I). Indeed, plasma insulin levels were significantly reduced by 44.8% ± 5.9% at baseline (*P* = 0.003) and by 42.7% ± 8.3% 15 minutes after an oral glucose bolus (*P* = 0.006) in *Ldlr*^–/–^ *Ndst1*^fl/fl^ *Alb-Cre*^+^ mice treated with the apoC-III ASO (Figure 6B). Furthermore, apoC-III ASO improved insulin sensitivity as shown by insulin tolerance test compared with control ASO–treated mice (Figure 6E). Overall, the increased insulin sensitivity was associated with improved glucose tolerance (Figure 6H). In contrast, apoC-III ASO intervention in *Apoe*^–/–^ *Ndst1*^fl/fl^ *Alb-Cre*^+^ and *Ldlr*^–/–^ *Lrp1*^fl/fl^ *Alb-Cre*^+^ mice did not improve plasma insulin levels (Figure 6, A and C), insulin sensitivity, (Figure 6, D and F), or glucose tolerance (Figure 6, G and I). Although apoC-III ASO reduced plasma triglyceride levels in both *Apoe*^–/–^ *Ndst1*^fl/fl^ *Alb-Cre*^+^ and *Ldlr*^–/–^ *Ndst1*^fl/fl^ *Alb-Cre*^+^ mice, it is important to note that insulin sensitivity was only improved in *Ldlr*^–/–^ *Ndst1*^fl/fl^ *Alb-Cre*^+^ mice, in which apoC-III ASO–mediated triglyceride lowering was achieved by improved TRL clearance compared with LPL activity in *Apoe*^–/–^ *Ndst1*^fl/fl^ *Alb-Cre*^+^ mice.

Next, we analyzed if changes in plaque inflammation or liver ER stress markers were associated with improved TRL clearance and insulin sensitivity in *Ldlr*^–/–^ *Ndst1*^fl/fl^ *Alb-Cre*^+^ mice, to explain the decrease in necrotic core formation. We found that proinflammatory genes, such as *interleukin 6* (*Il6*) and *tumor necrosis factor alpha* (*Tnfa*), were reduced, though nonsignificantly due to a high variability, in the plaque and liver, respectively (Figure 6, J and K). However, apoC-III lowering reduced ER stress in the liver, as determined by a 60.9% ± 17.2% reduction (*P* = 0.025) in *DNA damage inducible transcript 3* (*Ddit3*) and a 62.2% ± 15.1% reduction (*P* = 0.15) in *activating transcription factor 4* (*Atf4*) (Figure 6K). It was recently observed that apoC-III lowering benefits atherosclerosis due to a reduction in macrophage foam cell generation, which could explain the reduction in necrotic core formation (36). After 8 weeks of ASO treatment, we isolated peritoneal macrophages from *Ldlr*^–/–^ *Ndst1*^fl/fl^ *Alb-Cre*^+^ mice to determine in vivo foam cell formation by oil red O staining and cellular cholesteryl ester analysis (Supplemental Figure 5A). Lowering apoC-III with ASOs did not affect foam cell formation or the concentration of total cholesterol (TC), free cholesterol (FC), and cholesteryl ester (CE) in isolated peritoneal macrophages (Figure 6L). Moreover, gene expression of cholesterol efflux markers ATP binding cassette subfamily A member 1 and G member 1 (*Abca1* and *Abcg1*) was similar in atherosclerotic plaques from mice treated with control ASO or apoC-III ASO (Supplemental Figure 5, B and C). Overall, the data suggest that improved insulin sensitivity in *Ldlr*^–/–^ *Ndst1*^fl/fl^ *Alb-Cre*^+^ mice is associated with a decrease in necrotic core formation, inflammation, and ER stress.

### ApoC-III ASO and cholesterol-low diet intervention after Western diet feeding result in additive triglyceride lowering.

**Figure 7:**
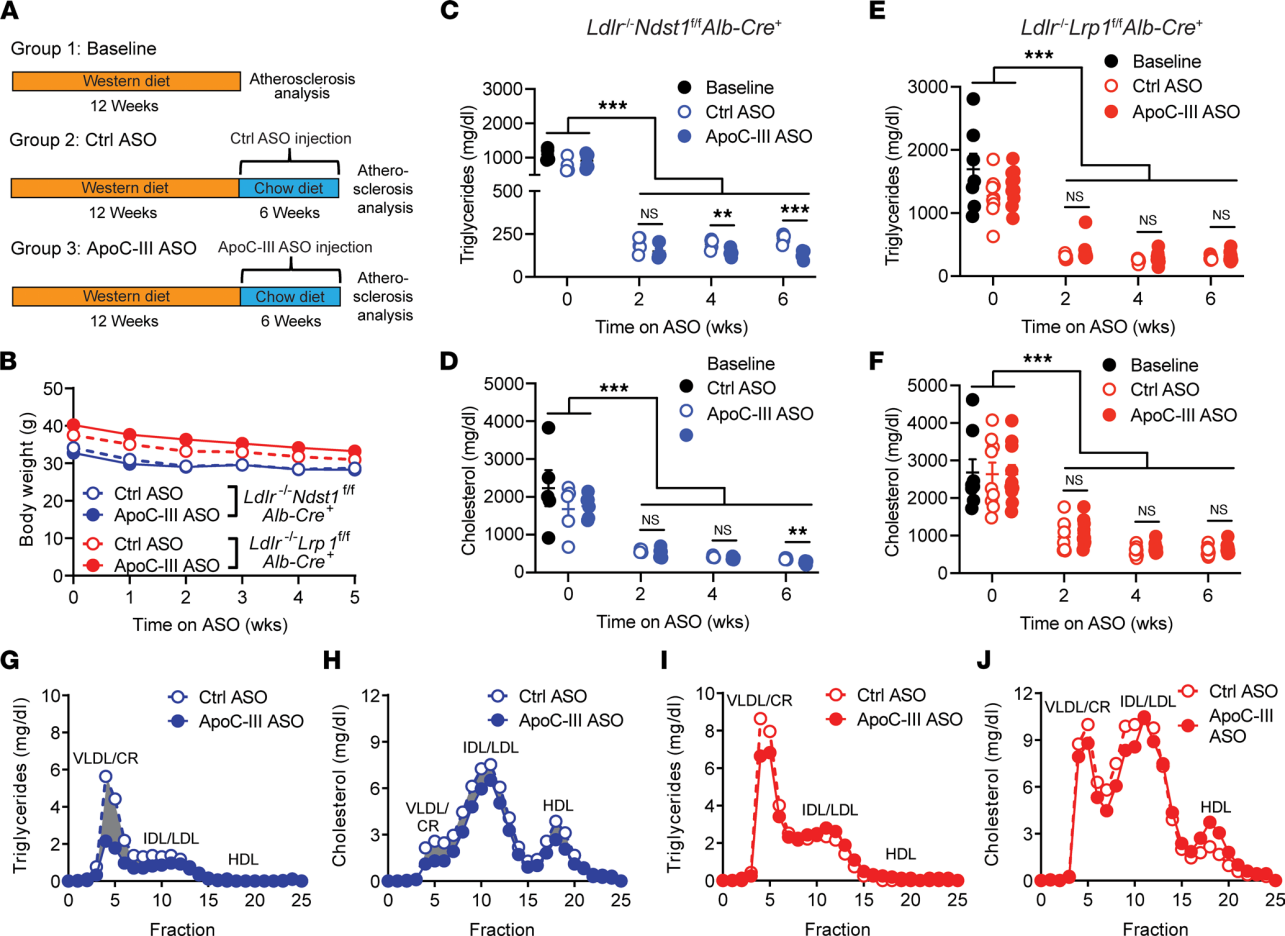
Diet intervention combined with apoC-III targeting reduces plasma lipid levels in mice with increased TRL clearance. (**A**) Atherosclerosis was induced in *Ldlr*^–/–^ *Ndst1*^fl/fl^ *Alb*-*Cre*^+^ and *Ldlr*^–/–^ *Lrp1*^fl/fl^ *Alb*-*Cre*^+^ mice by Western diet feeding for 12 weeks. Mice were then switched to a chow diet in combination with intraperitoneal injection of control ASO or apoC-III ASO (50 mg/kg body weight). Baseline data were evaluated after 12 weeks of Western diet. (**B**) Body weight of *Ldlr*^–/–^ *Ndst1*^fl/fl^ *Alb*-*Cre*^+^ (blue, *n* = 5–6/group) and *Ldlr*^–/–^ *Lrp1*^fl/fl^ *Alb*-*Cre*^+^ mice (red, *n* = 8–10/group). (**C**–**F**) Fasting plasma triglyceride and cholesterol levels in (**C** and **D**) *Ldlr*^–/–^ *Ndst1*^fl/fl^ *Alb*-*Cre*^+^ (*n* = 5–6/group) and (**E** and **F**) *Ldlr*^–/–^ *Lrp1*^fl/fl^ *Alb*-*Cre*^+^ mice (*n* = 8–10/group). (**G**–**J**) Size-exclusion FPLC analysis of pooled plasma samples (100 μL, *n* = 5/pool) to determine (**G** and **I**) triglycerides and (**H** and **J**) cholesterol in chylomicron remnant/VLDL, IDL/LDL, and HDL fractions of *Ldlr*^–/–^ *Ndst1*^fl/fl^ *Alb*-*Cre*^+^ and *Ldlr*^–/–^ *Lrp1*^fl/fl^ *Alb*-*Cre*^+^ mice, respectively. Data presented as mean ± SEM. Statistical differences between 2 groups were calculated using an unpaired 2-tailed Student’s *t* test. **P < 0.01, ***P < 0.001.

After diagnosis of a CVD event or detection of a significant CVD risk factor, such as elevated LDL-cholesterol, patients typically receive pharmacological treatment to lower LDL-cholesterol (statins or PCSK9 antibodies) and undergo a series of lifestyle changes, including low-cholesterol diets (30). Therefore, we set out to study the impact of apoC-III targeting on atherosclerosis using a combined approach of apoC-III ASO treatment and a cholesterol-lowering intervention, as this better resembles the current therapeutic approach. We generated a model where we induced atherosclerosis by feeding a Western diet for 12 weeks. After the initial atherogenic priming, we then intervened therapeutically by switching to a low-cholesterol diet (standard mouse chow) in conjunction with apoC-III ASO or control ASO administration for an additional 6 weeks (Figure 7A). The additional benefit of this model is that we can assess the impact of apoC-III lowering on established atherosclerotic lesions without the confounding continued high-cholesterol diet–driven expansion of lesions. To assess the impact of the diet switch and apoC-III ASO treatment, we determined atherosclerosis development after 12 weeks of Western diet to generate the baseline group. We focused on *Ldlr*^–/–^ *Ndst1*^fl/fl^ *Alb-Cre*^+^ mice to analyze the impact of apoC-III ASO–mediated TRL clearance on atherogenesis and *Ldlr*^–/–^ *Lrp1*^fl/fl^ *Alb-Cre*^+^ mice to assess effects independent of triglyceride lowering. After switching from the cholesterol-rich Western diet to a cholesterol-poor standard diet, body weight in both mouse groups declined; however, no differences were seen between mice treated with control ASO or apoC-III ASO (Figure 7B). As expected, the low-cholesterol diet significantly reduced plasma cholesterol and triglyceride levels by approximately 66% and approximately 75%, respectively, compared with baseline values in both hyperlipidemic mouse models (Figure 7, C–F). The reduction was observed as early as 2 weeks into the intervention, at which point maximal lipid reduction was achieved throughout the 6 weeks. In *Ldlr*^–/–^ *Ndst1*^fl/fl^ *Alb-Cre*^+^ mice, apoC-III ASO treatment induced an additional plasma triglyceride and cholesterol lowering (Figure 7, C and D). Triglyceride levels were significantly reduced by 26.5% ± 4.3% (*P* = 0.008) after 4 weeks and by 40.3% ± 4.2% (*P* < 0.001) after 6 weeks of apoC-III ASO compared with control ASO (Figure 7C). Also, plasma cholesterol levels were reduced by 25.0% ± 4.5% (*P* = 0.002) but only after 6 weeks of apoC-III ASO treatment (Figure 7D). The apoC-III ASO–mediated decrease in plasma triglycerides was caused by a reduction in the VLDL/chylomicron remnant fraction as analyzed by FPLC (Figure 7G), whereas reduced VLDL/chylomicron remnants and IDL/LDL, but also a reduction in HDL, contributed to reduced plasma cholesterol levels (Figure 7H). In contrast, apoC-III ASO did not affect plasma triglyceride (Figure 7, E and I) or cholesterol levels (Figure 7, F and J) compared to control ASO in *Ldlr*^–/–^ *Lrp1*^fl/fl^ *Alb-Cre*^+^ mice.

### Triglyceride lowering induced by combined apoC-III targeting and cholesterol-low diet intervention prevents atherosclerosis progression.

**Figure 8:**
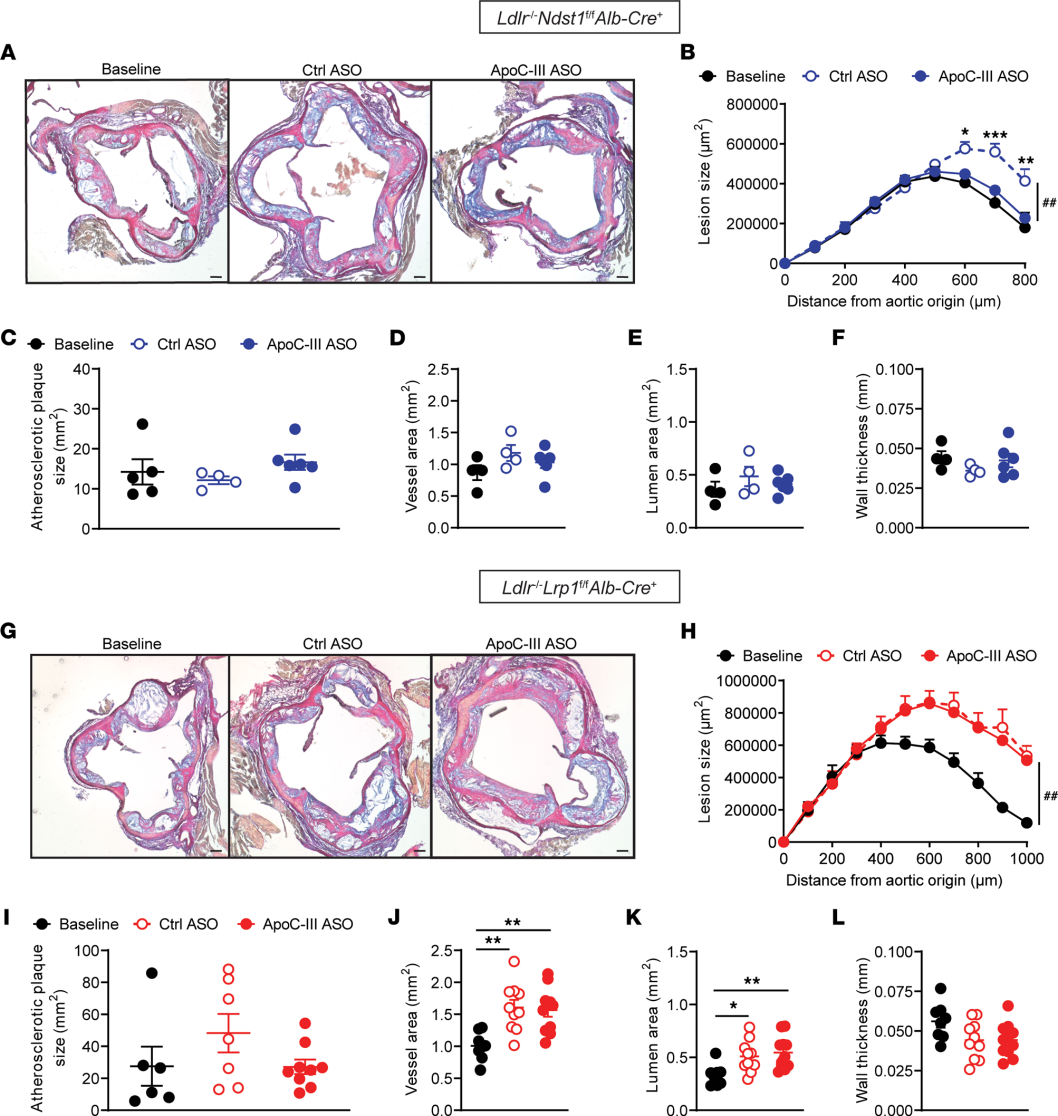
ApoC-III ASO–mediated lowering of plasma lipids improves atherosclerotic lesion size in diet intervention model. After diet intervention, (**A**–**F**) *Ldlr*^–/–^ *Ndst1*^fl/fl^ *Alb*-*Cre*^+^ (blue, *n* = 4–6/group) and (**G**–**L**) *Ldlr*^–/–^ *Lrp1*^fl/fl^ *Alb*-*Cre*^+^ mice (red, *n* = 6–10/group) were administered with control ASO or apoC-III ASO for 6 weeks and atherogenesis was analyzed. (**A** and **G**) Example images of aortic root cross sections stained with modified Verhoeff-van Gieson stain. (**B** and **H**) Quantification of atherosclerosis lesion size in aortic root cross sections. (**C** and **I**) En face analysis of the entire aorta using Sudan IV stain. Quantification of the (**D** and **J**) aortic vessel area, (**E** and **K**) aortic lumen area, and (**F** and **L**) vessel wall thickness. Data presented as mean ± SEM. Statistical differences in lesion size over distance were calculated using a 2-way ANOVA with Bonferroni’s post hoc analysis. Statistical differences between 3 groups were calculated using a 1-way ANOVA with Tukey’s post hoc analysis. *P < 0.05, **P < 0.01, ***P < 0.001; ^##^*P* < 0.01 compared with baseline.

We investigated if apoC-III targeting prevents progression of atherosclerosis after diet intervention by analyzing lesion sizes in the aortic root and whole aorta (Figure 8). In *Ldlr*^–/–^ *Ndst1*^fl/fl^ *Alb-Cre*^+^ mice receiving control ASO after the diet switch, the total lesion size further progressed compared with the baseline group (*P* < 0.001). Remarkably, diet intervention and apoC-III ASO treatment led to a significant decrease in aortic root lesion size compared with control ASO–treated *Ldlr*^–/–^ *Ndst1*^fl/fl^ *Alb-Cre*^+^ mice (*P* = 0.008, Figure 8, A and B). The lesion size was comparable with the baseline group, suggesting inhibition of plaque progression upon apoC-III ASO administration. No differences were observed in en face analysis (Figure 8C), aortic vessel area (Figure 8D), aortic lumen area (Figure 8E), and aortic wall thickness (Figure 8F). In contrast, atherosclerotic plaque analysis in aortic root cross sections (Figure 8, G and H) isolated from *Ldlr*^–/–^ *Lrp1*^fl/fl^ *Alb-Cre*^+^ mice revealed no differences between control ASO and apoC-III ASO. Compared with the baseline group levels, aortic root lesions significantly progressed to the same extent in both control ASO– and apoC-III ASO–treated mice (*P* < 0.001, Figure 8, G and H). No differences were observed in the en face aorta analysis (Figure 8I). Interestingly, both aortic vessel area (Figure 8J) and aortic lumen area (Figure 8K) were increased in mice on control ASO or apoC-III ASO, respectively, compared with the baseline group, indicating an ongoing outward remodeling of the plaque. No changes were seen in the thickness of the aortic wall (Figure 8L). The data indicate that additional triglyceride lowering mediated by apoC-III lowering after diet intervention prevents further atherosclerosis development.

### ApoC-III ASO in conjunction with a low-cholesterol diet switch improves plaque remodeling and markers of plaque stability.

**Figure 9:**
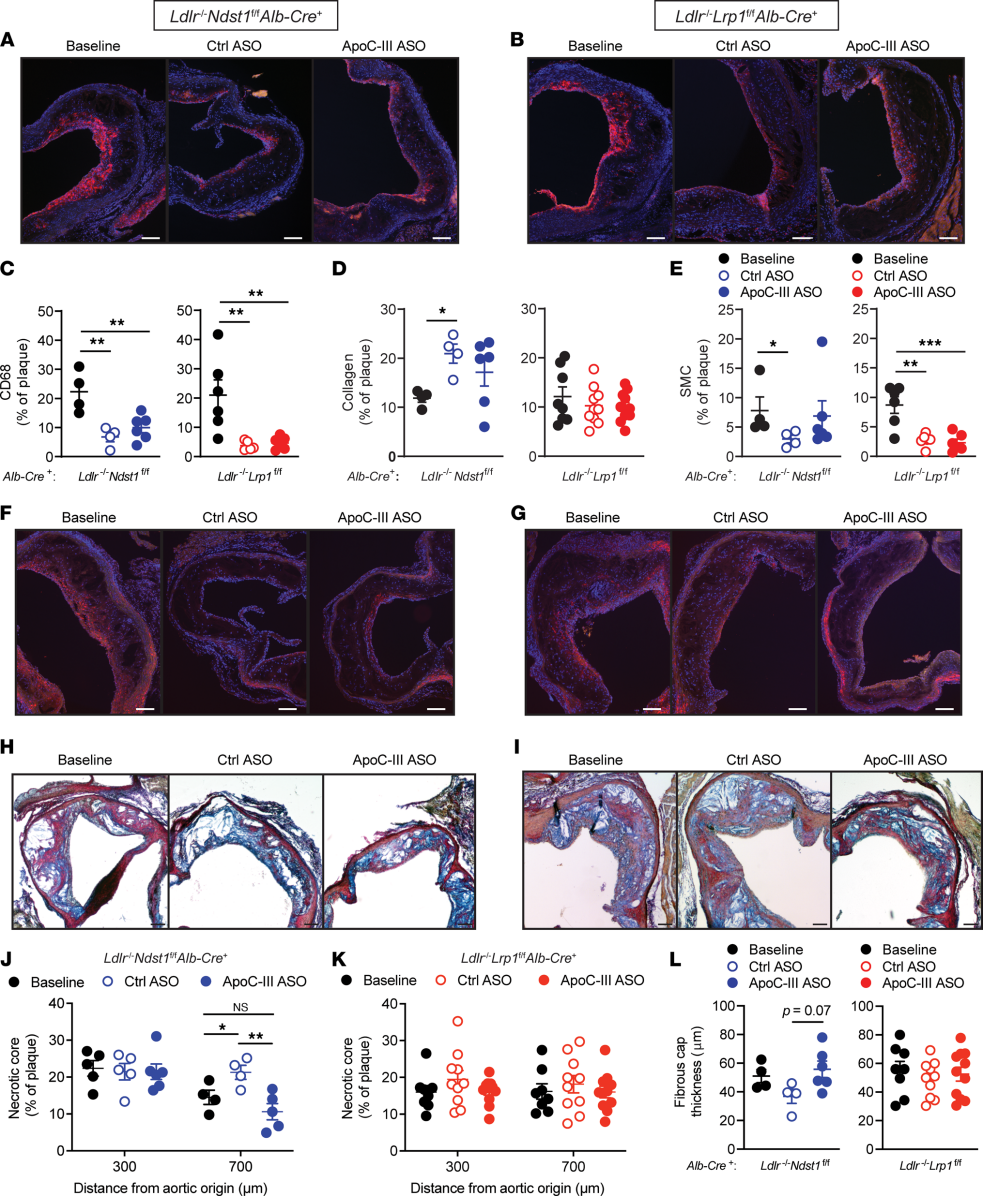
Histological quantification of aortic lesions after diet intervention. After 12 weeks of Western diet feeding followed by 6 weeks of chow diet feeding and control ASO or apoC-III ASO treatment, atherosclerotic lesions in *Ldlr*^–/–^ *Ndst1*^fl/fl^ *Alb*-*Cre*^+^ (blue) and *Ldlr*^–/–^ *Lrp1*^fl/fl^ *Alb*-*Cre*^+^ (red) mice were analyzed. For baseline determination, aortas were harvested from mice after 12 weeks of Western diet. (**A** and **B**) Representative images of aortic root cross sections (700 μm) stained with anti-CD68 antibody. (**C**) Quantification of CD68 staining (*n* = 4–6/group). (**D**) Collagen quantification with modified Verhoeff-van Gieson stain (*n* = 4–10 group). (**E**) Quantification of anti-SMC immunohistochemistry (*n* = 4–6/group). (**F** and **G**) Representative images of SMC detection in (**F**) *Ldlr*^–/–^ *Ndst1*^fl/fl^ *Alb*-*Cre*^+^ and (**G**) *Ldlr*^–/–^ *Lrp1*^fl/fl^ *Alb*-*Cre*^+^ mice. (**H**–**K**) Analysis of necrotic core area in (**H** and **J**) *Ldlr*^–/–^ *Ndst1*^fl/fl^ *Alb*-*Cre*^+^ and (**I** and **K**) *Ldlr*^–/–^ *Lrp1*^fl/fl^ *Alb*-*Cre*^+^ mice (*n* = 4–10/group). Data were quantified as a percentage of the plaque size. (**L**) Quantification of fibrous cap (*n* = 4–11/group). Scale bars equal 100 μm. Data presented as mean ± SEM. Statistical differences between 2 groups were calculated using an unpaired 2-tailed Student’s *t* test and between 3 groups were calculated using a 1-way ANOVA with Tukey’s post hoc analysis. *P < 0.05, **P < 0.01, ***P < 0.001.

We characterized if composition of atherosclerotic lesions in the diet intervention model was affected by additional apoC-III triglyceride lowering as in the previous observations (Figure 5). Macrophage content, assessed by CD68 stain, was significantly reduced in both control ASO– and apoC-III ASO–treated groups compared with baseline (Figure 9, A–C). This reduction in macrophage lesion content after the low-cholesterol diet switch was observed in both *Ldlr*^–/–^ *Ndst1*^fl/fl^ *Alb-Cre*^+^ and *Ldlr*^–/–^ *Lrp1*^fl/fl^ *Alb-Cre*^+^ mice, indicating significant lesion remodeling. ApoC-III inhibition did not additionally affect CD68-positive area (Figure 9C). Further, analysis of the collagen area in aortic lesions of *Ldlr*^–/–^ *Ndst1*^fl/fl^ *Alb-Cre*^+^ mice showed no differences between the apoC-III ASO group and baseline, whereas the control ASO group had significantly more collagen compared with baseline (Figure 9D). No differences in collagen were observed between apoC-III–treated mice and control ASO (*P* = 0.28) or baseline (*P* = 0.14) in mice lacking *Ldlr* and *Lrp1*. SMC content was significantly reduced by 2.3-fold in lesions from *Ldlr*^–/–^ *Ndst1*^fl/fl^ *Alb-Cre*^+^ mice on control ASO compared with baseline (*P* = 0.03). In contrast, no significant loss of SMC content was observed in the apoC-III ASO–treated group when compared to baseline measurements (Figure 9, E–G). In *Ldlr*^–/–^ *Lrp1*^fl/fl^ *Alb-Cre*^+^ mice, the SMC content in the aortic root was reduced by 68.6% ± 5.1% (*P* = 0.001) and 73.5% ± 7.0% (*P* < 0.001) compared with baseline in control ASO– and apoC-III ASO–treated mice, respectively (Figure 9E). Analysis of oxPL content (Supplemental Figure 6, A–C) and apoptotic cells using TUNEL stain (Supplemental Figure 6, D–F) did not show any significant alteration induced by the diet or apoC-III targeting. To further analyze the impact of apoC-III inhibition on the progression of advanced atherosclerosis, we analyzed the necrotic core area in aortic root cross sections (Figure 9, H–K). After diet intervention, *Ldlr*^–/–^ *Ndst1*^fl/fl^ *Alb-Cre*^+^ mice receiving control ASO showed a significant progression toward larger necrotic cores (+46.7% ± 13.0%, *P* = 0.046) in aortic root cross sections taken at 700 μm from the aortic origin compared with baseline. In contrast, apoC-III ASO–mediated reduction in plasma lipid levels reduced necrotic core area by 50.1% ± 10.3% (*P* = 0.004) compared with control ASO. This apoC-III ASO–mediated reduction in necrotic core was only observed at cross sections taken 700 μm from the aortic origin (Figure 9J). In contrast, in *Ldlr*^–/–^ *Lrp1*^fl/fl^ *Alb-Cre*^+^ mice, no changes in necrotic core area were observed (Figure 9, I and K). Fibrous caps were thicker in apoC-III ASO–treated *Ldlr*^–/–^ *Ndst1*^fl/fl^ *Alb-Cre*^+^ mice compared with the control ASO group, but no differences were observed in the *Ldlr*^–/–^ *Lrp1*^fl/fl^ *Alb-Cre*^+^ intervention group (Figure 9L). Necrosis of lesion foam cells is the result of an imbalance between cholesterol influx and efflux. Reverse cholesterol transport plays an important role in preventing the progression of atherosclerosis necrosis by facilitating cholesterol efflux via 2 ATP binding cassette transporters, ABCA1 and ABCG1 (37, 38). Lesion transcript analysis revealed that apoC-III ASO–mediated lowering of plasma lipids in *Ldlr*^–/–^ *Ndst1*^fl/fl^ *Alb-Cre*^+^ mice increased expression of both *Abca1* (37.3% ± 9.1%, *P* = 0.056) and *Abcg1* (77.7% ± 16.2%, *P* = 0.03) compared with control ASO mice (Supplemental Figure 6G). Gene expression of inflammatory markers (*F4-80*, *Il6*, *Il10*, and *Tnfa*) and the apoptosis marker *Casp3* were not affected by apoC-III ASO (Supplemental Figure 6G). Thus, inhibition of apoC-III combined with diet intervention prevents the progression of atherosclerotic lesions and results in lesion remodeling as shown by a significant reduction in necrotic core area and maintenance of the SMC cap content when the apoC-III ASO mediated improvements in TRL clearance.

## Discussion

Findings from this study specifically addressed the question of whether apoC-III–mediated HTG or apoC-III–mediated inflammatory processes, or both, contribute to atherogenesis. Our data suggest that (i) apoC-III inhibition increases the stability of vulnerable plaques as shown by a reduction in necrotic core area and increase in SMC content and fibrous cap thickness, (ii) this effect is dependent on triglyceride lowering via improved TRL clearance, (iii) increased insulin sensitivity is associated with reduced necrotic core area and increased SMC content, and (iv) apoC-III deficiency in conjunction with diet intervention prevents the progression of atherosclerosis. These potentially novel conclusions could only be obtained by using our set of mice in which targeting apoC-III with ASOs decreased plasma triglyceride levels via either enhanced LPL activity (*Apoe*^–/–^ *Ndst1*^fl/fl^ *Alb-Cre*^+^) or accelerated hepatic TRL clearance (*Ldlr*^–/–^ *Ndst1*^fl/fl^ *Alb-Cre*^+^) or in mice in which apoC-III ASO did not affect plasma triglyceride levels at all (*Ldlr*^–/–^ *Lrp1*^fl/fl^ *Alb-Cre*^+^). *Ldlr*^–/–^ *Lrp1*^fl/fl^ *Alb-Cre*^+^ mice are an especially powerful model. These mice are the only genetic rodent model available to our knowledge in which apoC-III targeting does not lower plasma lipid levels, allowing us to study effects of apoC-III atherosclerosis independent of triglyceride lowering, such its effects on inflammation.

Targeting apoC-III mRNA with antisense strategy has emerged as a promising triglyceride-lowering drug with the goal to reduce the increased risk of acute pancreatitis in those with severe HTG and to reduce the risk of CVD in those with moderate HTG. (28). Yet, it remains unknown if interventional apoC-III inhibition as opposed to lifelong lowering (39) improves the atherosclerotic burden in humans and animal models (40). Thus, we set out to explore the impact of lowering apoC-III under various feeding conditions. Both *apoE*- and *Ldlr*-deficient mice are commonly used as atherosclerosis models and are known to develop moderate lesions on a chow diet (41, 42). By combining those models with hepatic TRL receptor inactivation, e.g., HSPG receptor or LRP1, plasma lipid levels are additionally increased compared with the single *Apoe* or *Ldlr* knockout models (11, 43), allowing plaque development on a cholesterol-poor diet. However, on a standard cholesterol-poor diet, no differences in lesion size and area were observed after apoC-III ASO treatment compared to control ASO. We reasoned that the plaques were early-type lesions consisting mainly of foam cells due to low lipid levels and that the apoC-III–mediated reduction in triglyceride levels was not sufficient to measure an impact of apoC-III on early atherosclerotic lesion development. We then fed the mice a Western diet (42% kcal from fat, 0.15% cholesterol), which induced more advanced lesions (42, 44, 45). Although apoC-III knockdown significantly increased measures of plaque stability, lesion size and volume were not altered despite substantial triglyceride lowering and even a slight reduction in cholesterol. There are multiple ways to explain this unexpected result. First, Western diet feeding led to severe hypercholesterolemia with cholesterol levels over 1000 mg/dL in *Apoe*^–/–^ *Ndst1*^fl/fl^ *Alb-Cre*^+^ and in *Ldlr*^–/–^ *Ndst1*^fl/fl^ *Alb-Cre*^+^ mice and beyond 2000 mg/dL in *Ldlr*^–/–^ *Lrp1*^fl/fl^ *Alb-Cre*^+^ mice. These extremely elevated plasma cholesterol levels will likely have obscured the antiatherogenic effect of apoC-III–mediated triglyceride lowering. Second, the apoC-III ASO–mediated reduction in triglycerides is mainly explained by a decrease in the TRL remnant fraction with only marginal changes in the IDL and LDL fraction (11, 17). Initial steps in atherosclerosis comprise the penetration of LDL particles into the vessel wall. However, larger particles, such as chylomicron remnants and VLDL, cannot enter the endothelium as efficiently as smaller atherogenic LDL particles (46–48). Hence, a reduction in chylomicron remnants and VLDLs is not directly associated with improved atherogenesis. This was reported to occur in mice where inactivation of LPL resulted in more circulating chylomicron remnants and VLDL without affecting atherogenesis (48). Third, we treated the mice with ASOs for only 8 weeks to get insights about the short-term effects of therapeutically apoC-III targeting on atherosclerosis. This is in strong contrast to individuals who benefit from lifelong atheroprotective effects of apoC-III loss-of-function mutation (9, 25, 26). It may be that long-term treatment with ASOs against apoC-III reduces lesion size.

It was rather unexpected to see no differences in lesion size when lowering apoC-III with ASOs given the atheroprotective effect of apoC-III loss-of-function mutations in humans has been described over a huge variety of studies (25–27, 49). However, unlike the other reports, in the current study plasma triglycerides were lowered starting at a high baseline for only a short period (8–12 weeks). It is worth mentioning that a recent study analyzed atherogenesis in *Apoc3*-KO mice crossed into an *Ldlr*^–/–^ background (50). Interestingly, no differences in lesion size were reported between *Apoc3*^–/–^ *Ldlr*^–/–^ and *Ldlr*^–/–^ mice while *Apoc3*-transgenic mice crossed into an *Ldlr*^–/–^ background experienced increased atherosclerotic plaque development (50) as described before (51). Similarly, apoC-III inhibition in Western diet feeding of diabetic mice did not reduce atherosclerotic lesion but only necrotic core area, which is in line with our study (36). Importantly, in the same study, it is reported that apoC-III ASO could prevent diabetes-induced atherosclerosis when feeding a low-cholesterol diet (36). However, in this model, both plasma triglycerides and cholesterol were drastically reduced.

To further investigate the role of apoC-III on atherogenesis, we then analyzed plaque formation in a diet intervention model. We found that a combination of apoC-III inhibition and chow diet feeding after inducing atherosclerosis by Western diet prevented the progression of atherosclerosis. This is an important finding as it suggests that therapeutic apoC-III inhibition can reduce CVD risk. Of note, in this model apoC-III not only reduced triglycerides but also reduced cholesterol levels. However, a dual therapeutic approach would be most advantageous by targeting apoC-III combined with statins and diet intervention.

In advanced atherosclerotic lesions, plaque stability plays a key role in the occurrence of cardiovascular events as plaque rupture results in the recruitment of platelets and blood coagulation, ultimately forming a thrombus (30, 31). The formation of necrotic cores predisposes to plaque rupture (31, 52). Analysis of the necrotic core area revealed major differences between our models. ApoC-III lowering resulted in a significant reduction in necrotic core area in *Apoe*^–/–^ *Ndst1*^fl/fl^ *Alb-Cre*^+^ and in *Ldlr*^–/–^ *Ndst1*^fl/fl^ *Alb-Cre*^+^ mice. In these models apoC-III lowering improves tissue LPL activity or TRL clearance, respectively (11, 17). This contrasts with *Ldlr*^–/–^ *Lrp1*^fl/fl^ *Alb-Cre*^+^ mice, in which plasma triglyceride levels were not altered and necrotic core area was similar between control and apoC-III ASO treatment. The data demonstrate that targeting apoC-III improves measures of plaque stability when lipid lowering is achieved by the apoC-III ASO, though it is not entirely understood if this the associated reduction in lesion size and stability is solely driven by accelerated TRL clearance. Nevertheless, our results are in line with data by Kanter et al., as they showed that necrotic core formation was reduced in diabetic mice treated with apoC-III ASO (36). Also in this model, triglyceride and cholesterol lowering was achieved after administration of the apoC-III ASO. Macrophage apoptosis is a result of ER stress, which is induced by multiple factors including insulin resistance, oxPL, TRLs, and saturated fatty acids (32). Tall and colleagues showed that the deficiency of the macrophage insulin receptor results in an altered ER stress response by diminished phosphorylation of serine-threonine protein kinase AKT, an antiapoptotic protein. Consequently, ER stress induces expression of scavenger receptors and stimulates apoptosis of macrophages (53). Hence, insulin resistance may lead to necrotic core formation through the induction of apoptosis (54).

In our study, we found that lowering apoC-III not only reduced plasma triglycerides but also decreased plasma insulin levels and improved insulin sensitivity in *Ldlr*^–/–^ *Ndst1*^fl/fl^ *Alb-Cre*^+^ mice treated with apoC-III ASOs. In contrast, in *Ldlr*^–/–^ *Lrp1*^fl/fl^ *Alb-Cre*^+^ mice, targeting apoC-III did not alter plasma triglyceride levels, glucose homeostasis, or necrotic core formation. Improved insulin sensitivity did not correlate with reduced triglyceride levels overall in our study. However, it is important to appreciate that the mechanism driving the apoC-III ASO–mediated reduction in plasma triglyceride levels differs between the 2 models where apoC-III ASOs reduced plasma triglyceride levels. In *Apoe*^–/–^ *Ndst1*^fl/fl^ *Alb-Cre*^+^ mice, the apoC-III ASO lowers triglycerides by promoting LPL activity in adipose tissue and not by promoting hepatic TRL clearance. In contrast, apoC-III ASO reduces circulating triglyceride levels in *Ldlr*^–/–^ *Ndst1*^fl/fl^ *Alb-Cre*^+^ mice by promoting hepatic TRL clearance. Thus, the improvement in insulin sensitivity may be associated with a reduction in circulating TRL particles rather than with a size reduction of TRLs induced by LPL remodeling. Future studies are needed to confirm that this association is correct. Another possible explanation relates to the degree of insulin resistance, which differs between the models, with the *Ldlr*^–/–^ *Ndst1*^fl/fl^ *Alb-Cre*^+^ mice having the most significant insulin resistance phenotype. It could well be that the degree of insulin resistance is not far enough advanced in *Apoe*^–/–^ *Ndst1*^fl/fl^ *Alb-Cre*^+^ mice to observe any improvement due to apoC-III ASO–induced triglyceride lowering. Possibly longer feeding regimens could address this in future studies.

The data suggest that, if triglyceride lowering is achieved via improved TRL clearance, apoC-III deficiency enhances insulin signaling and therewith might resolve ER stress, resulting in smaller necrotic cores. This hypothesis is supported by recent studies that found an increase in inflammation and ER stress in *Apoc3*-transgenic mice (55). Moreover, Botteri et al. suggested that apoC-III can promote ER stress and insulin resistance by ERK1/2 activation through TLR2 in vitro (56). We found that the ER stress markers *Ddit3* and *Atf4*, both activators of the unfolded protein response (57, 58), were downregulated in the liver, but not in plaques of *Ldlr*^–/–^ *Ndst1*^fl/fl^ *Alb-Cre*^+^ mice. The decrease in ER stress seems unassociated with alterations in hepatic lipid content, as no differences in hepatic lipid levels were measured under HFD feeding as reported before (11, 17). Although we found profound evidence that apoC-III lowering improves measures of plaque stability by reducing plasma triglycerides and improving insulin sensitivity, one must be mindful when translating those results to humans (42, 44). Further studies are essential to fully understand the molecular mechanism of how apoC-III–mediated triglyceride lowering and improved insulin sensitivity affect necrotic core formation.

Previous reports support that apoC-III can promote a sterile inflammation in macrophages (22). We did not observe significant changes in lesion inflammation after apoC-III lowering. However, we performed a basic analysis of the inflammatory status. Therefore, we cannot entirely exclude that some of the effects we see in plaque remodeling are, to some extent, due to attenuated inflammation induced by lower circulating apoC-III levels. Despite an overall improvement in plaque stability markers, such as increased SMC content, we did not observe a reduction in macrophage content in the lesions after apoC-III ASO intervention. We measured macrophage content by CD68 staining, which is also expressed by lesion SMCs (59). Hence, it is conceivable that we did have a reduction in macrophage content in apoC-III ASO–treated *Ldlr*^–/–^ *Ndst1*^fl/fl^ *Alb-Cre*^+^ mice but that it got obscured by the increased CD68-positive SMCs. We also did not address potential changes in the inflammatory transcriptome of circulating monocytes in the apoC-III ASO–treated groups. Hence, it will be relevant to perform refined epigenetic and transcriptomic analyses in circulating monocytes, lesion macrophages, and other inflammatory cells in future experiments. Applying single-cell RNA sequencing and ChIP sequencing will help to understand better the relative contribution of apoC-III’s impact on inflammation during atherogenesis and lesion remodeling.

In conclusion, we show that lowering apoC-III with ASOs results in significant plaque remodeling when plasma triglycerides are reduced. More importantly, our data support the concept that apoC-III ASO treatment in conjunction with cholesterol lowering and diet intervention can further halt progression of atherosclerosis and advanced plaque rupture, providing an additional treatment paradigm for patients who have HTG and increased CVD risk.

## Methods

### Study design.

The objective of this study was to assess the impact of apoC-III inhibition using ASOs on atherosclerosis. In our study, we made use of 3 mouse models we previously characterized (11, 17), which differ in their response to apoC-III ASO. ApoC-III lowering with ASOs reduced plasma triglyceride levels in *Apoe*^–/–^ *Ndst1*^fl/fl^ *Alb-Cre*^+^ and *Ldlr*^–/–^ *Ndst1*^fl/fl^ *Alb-Cre*^+^ mice, and thus, enabled us to determine if apoC-III ASO–mediated triglyceride lowering improves atherogenesis. However, in the first model, improved tissue LPL activity mediated the reduction in plasma triglyceride, while in the latter model apoC-III inhibition increased hepatic TRL clearance via LRP1. In the third model, *Ldlr*^–/–^ *Lrp1*^fl/fl^ *Alb-Cre*^+^, plasma triglyceride levels were unchanged upon apoC-III ASO treatment due to the lack of LDLR and LRP1. Hence, this model allows us to study whether apoC-III inhibition alters inflammation and consequently improves atherosclerosis independently of TRL lowering. At the beginning of the study, littermates were randomly assigned to either control ASO or apoC-III ASO, and the efficiency of the apoC-III inhibition was assessed by measuring plasma triglycerides throughout the experiment as well as by gene expression study and Western blot at the predetermined endpoint. Sample sizes were determined based on experiments published previously (34, 60) and are indicated in the figure legends. No blinding was performed during experimental administering of the ASOs to the mice. However, the investigators were blinded to the treatment groups for all downstream experiments, except the Western blot, and quantification of the data using mouse ID numbers without revealing the treatment until after the analysis.

### Mice.

*Apoe*^–/–^, *Ldlr*^–/–^, *Lrp1*^fl/fl^, and *Alb-Cre*^+^ mice were purchased from The Jackson Laboratory. *Ndst1*^fl/fl^ *Alb-Cre*^+^, *Apoe*^–/–^ *Ndst1*^fl/fl^ *Alb-Cre*^+^, *Ldlr*^–/–^ *Ndst1*^fl/fl^ *Alb-Cre*^+^, and *Ldlr*^–/–^ *Lrp1*^fl/fl^ *Alb-Cre*^+^ mice were generated and genotyped as described (43, 61, 62). All animals were fully backcrossed on C57BL/6 background. All animals were housed and bred in vivaria approved by the Association for Assessment and Accreditation of Laboratory Animal Care located in the School of Medicine, UCSD, following standards and procedures approved by the UCSD Institutional Animal Care and Use Committee. Male mice were weaned at 4 weeks, maintained on a 12-hour light cycle, and fed ad libitum with water and standard rodent chow (PicoLab Rodent Diet 20 5053) or a Western diet (TD.88137, Envigo Teklad) containing 42% kcal from fat. Mice received ION 440726 (murine apoC-III ASO) or ION 141923 (murine control ASO) at 50 mg/kg/w (Supplemental Table 1) via intraperitoneal injections.

### RNA analysis.

Total RNA was isolated in TRIzol (Invitrogen) from homogenized tissue and cells and purified using RNeasy columns and RNase-free DNase digestion according to the manufacturer’s instructions (QIAGEN). The quality and quantity of the total RNA were monitored and measured with a NanoDrop (NanoDrop Technologies, Inc) following the manufacturer’s instructions. For quantitative PCR analysis, 1 μL of cDNA was used for real-time PCR with gene-specific primers (Supplemental Table 2) and TBP as a housekeeping gene on a BioRad CFX96 Real-time PCR system.

### TRL analysis.

TRLs were analyzed by SDS-PAGE on 4%–12% Bis-Tris gradient gels (NuPage, Invitrogen). Proteins were visualized by silver staining (Pierce) or after transfer to Immobilon-FL PVDF membranes (MilliporeSigma). Membranes were blocked with Odyssey Blocking Buffer (LI-COR Biosciences) for 30 minutes and incubated overnight at 4°C with respective antibodies. Goat, mouse, and rabbit antibodies were incubated with secondary Odyssey IR dye antibodies (1:14000) and visualized with an Odyssey IR Imaging system (LI-COR Biosciences). Western blot primary antibodies included rabbit anti-mouse apoB (Abcam ab20737, 1:1000), rabbit anti-mouse apoC-III (Ionis Pharmaceuticals, 1:2000) (63), and rabbit anti-mouse apoE (Meridian Life Sciences, K23100R, 1:1000).

### Lipid analysis.

Lipid levels were analyzed in plasma and liver samples as previously described (11, 64). Blood was drawn via the tail vein from mice fasted for 5 hours. Total plasma cholesterol and plasma triglyceride levels were determined using commercially available kits (Sekisui Diagnostics).

### Quantification of atherosclerotic lesions.

Fasted *Apoe*^–/–^ *Ndst1*^fl/fl^ *Alb-Cre*^+^, *Ldlr*^–/–^ *Ndst1*^fl/fl^ *Alb-Cre*^+^, and *Ldlr*^–/–^ *Lrp1*^fl/fl^ *Alb-Cre*^+^ mice were perfused with 10 mL of PBS following cardiac puncture. The heart and ascending aorta down to the iliac bifurcation were removed and incubated in sucrose buffer or formalin, respectively. The hearts were processed and stained by an atherosclerosis core morphology group. The isolated hearts were sectioned by cutting several 10 μm paraffin cross sections starting with the first appearance of the first leaflet of the aortic valve until the last leaflet in 100 μm sections. Aortic root atherosclerosis was analyzed using modified Verhoeff-van Gieson elastic staining to enhance the contrast between the intima and surrounding tissues. At each 100 μm cross section, the mean lesion size in each mouse was analyzed by computer-assisted morphometry (Image-Pro Plus 6.3, Media Cybernetics) by 2 investigators following a blinded study protocol. After removal of adventitial tissues, the aortas were incised longitudinally, pinned flat, and stained for neutral lipids using Sudan IV. Images were acquired using a Leica MSV266 microscope with an attached Leica Ic80 HD camera. Vessel areas, lesion areas, lumen areas, and vessel wall thickness of blinded samples were measured using ImageJ analysis software (NIH).

### Histology of atherosclerotic lesions.

Immunohistochemical analyses were performed on sections of paraformaldehyde-fixed and paraffin-embedded tissues. Necrotic core formation and collagen content were analyzed on modified Verhoeff-van Gieson elastic–stained aortic root cross-sectional lesions (300 μm or 700 μm or from beginning of aortic root) of *Apoe*^–/–^ *Ndst1*^fl/fl^ *Alb-Cre*^+^, *Ldlr*^–/–^ *Ndst1*^fl/fl^ *Alb-Cre*^+^, and *Ldlr*^–/–^ *Lrp1*^fl/fl^ *Alb-Cre*^+^ mice by using ImageJ analysis software. To determine the relative necrotic core area per plaque, we measured the absolute necrotic area by planimetry and then calculated the relative necrotic core area by measuring the plaque area (necrotic core area/plaque area). Necrotic cores were defined as areas in which no extracellular matrix (total loss of collagen/blue stain) was detected and replaced by dead cells and cellular debris as indicated by absence or fragmentation of nuclei. The fibrous cap was determined as the minimal thickness of the fibrous tissue overlying the necrotic core. If multiple necrotic cores were present within 1 plaque, the thickness of all fibrous caps was determined, and the average was used for further analyses (65). Aortic root cross-sectional lesions (300 μm or 700 μm from beginning of aortic root) were further stained with anti-CD68 (ab125212, Abcam), anti-SMC (ab5694, Abcam), and biotinylated E06 (34) for 12 hours at 4°C to analyze macrophage infiltration, SMC content, and oxPL accumulation, respectively. TUNEL analysis was performed using an In Situ Cell Death Detection Kit (TMR red, Roche) as per kit manufacturer’s instructions and counterstained with DAPI (ThermoFisher). TUNEL-positive cells in aortic sinus were quantified to the total cell count as measured by DAPI stain. Slides were imaged using a Keyence BZ-X800, and blinded samples were analyzed using ImageJ analysis software.

### Metabolic studies.

Measurements of glucose metabolism were performed in *Apoe*^–/–^ *Ndst1*^fl/fl^ *Alb-Cre*^+^, *Ldlr*^–/–^ *Ndst1*^fl/fl^ *Alb-Cre*^+^, and *Ldlr*^–/–^ *Lrp1*^fl/fl^ *Alb-Cre*^+^ mice. First, a glucose tolerance test was performed to determine the efficiency of insulin to decrease fasting glucose levels. Therefore, mice were fasted for 5 hours and orally gavaged with 2 mg/g body weight glucose. Plasma glucose levels were measured in blood samples from the tail vein at baseline, 15 minutes, 30 minutes, 60 minutes, 90 minutes, and 120 minutes after gavage using an Accu-Check Nano glucose monitor system (Roche Applied Science GmbH). Plasma insulin levels were measured before and 15 minutes after a glucose gavage via the ultrasensitive mouse insulin ELISA kit (Chrystal Chem). Second, an insulin tolerance test was performed to evaluate the insulin sensitivity. An insulin solution (0.6 U/kg body weight) was intraperitoneally injected into fasted (5 hours) mice, and glucose levels were monitored as described above.

### Peritoneal macrophages.

To analyze the effect of apoC-III lowering with ASOs on inflammation, peritoneal macrophages were isolated from the peritoneal cavity of *Ldlr*^–/–^ *Ndst1*^fl/fl^ *Alb-Cre*^+^ mice after 8 weeks of ASO administration, and macrophage foam cell formation and secretion of inflammatory markers were measured. To isolate peritoneal macrophages, 7 mL of ice-cold DMEM-10% FCS and 2 mL of air were injected into the peritoneal cavity. After gently massaging the anterior and lateral walls of the abdomen, the fluid containing peritoneal macrophages was collected and placed on ice. The cells were centrifuged for 5 minutes at 300*g* and 4°C and cultured in DMEM containing 10% FCS and 100 U/mL penicillin/streptomycin. After 2 hours of incubation at 37°C, the media were aspirated, and the adherent cells were washed with PBS. Macrophage purity of the adherent cells was greater than 95% based on F4/80^+^ cells determined by flow cytometry. Macrophage CE content was quantitated immediately using the Amplex Red Cholesterol Assay Kit (ThermoFisher). CE content was calculated by subtracting FC from TC for each sample. Foam cell formation was analyzed by staining cultured peritoneal macrophages with oil red O stain and quantified by using ImageJ analysis software. Inflammatory markers were determined by quantitative PCR (see above, Supplemental Table 2).

### FPLC.

Plasma was pooled from several mice (100 μL per mouse, *n* = 3–5 mice per genotype) and separated by gel filtration FPLC using a GE Superose 6 10/300 GL column in 0.15 M sodium chloride containing 0.01 M disodium hydrogen phosphate and 0.2 mM ethylenediaminetetraacetic acid, pH 7.4. Fractions (0.5 mL) were collected (0.5 mL/min) and total cholesterol and triglyceride levels were determined enzymatically as described above.

### Statistics.

Statistical analyses were performed using GraphPad Prism 8 (GraphPad Software). Normality was tested via Shapiro-Wilk test, and *F* tests were performed to analyze equal variances. Data that passed both tests were analyzed by 2-tailed Student’s *t* test for 2-group comparisons and 1-way ANOVA for comparison of multiple groups (>2) followed by Tukey’s post hoc testing. For data with multiple variables, e.g., atherosclerotic lesion size over distance or glucose measurements over time, a 2-way ANOVA for repeated measurements followed by Bonferroni’s post hoc test or Fisher’s least significant difference post hoc testing was performed. All data are presented as mean ± SEM. *P* values less than 0.05 were considered significant.

### Study approval.

All mouse experiments were approved by the UCSD Institutional Animal Care and Use Committee.

## Author contributions

BR, JLW, and PLSMG conceived the study. BR, XS, and PLSMG developed methodology. BR, SP, XS, ARP, and GMD investigated. BR visualized data. BR and PLSMG acquired funding. PLSMG provided project administration. AEM, RGL, and RMC provided resources. PLSMG supervised. BR and PLSMG wrote the original draft. BR, XS, AEM, RGL, RMC, JLW, ST, and PLSMG reviewed and edited the draft.

## Supplementary Material

Supplemental data

## Version 1 06/02/2022

In-Press Preview

## Version 2 07/08/2022

Electronic publication
