# Supplementary material for: Interventional hepatic apoC-III knockdown improves atherosclerotic plaque stability and remodeling by triglyceride lowering
Source: JCI Insight. 2022 Jul 8;7(13):e158414. doi: 10.1172/jci.insight.158414 (PMC9310539; doi:10.1172/jci.insight.158414)
Supplement: Supplemental data [file jciinsight-7-158414-s034.pdf]

Supplementary Materials:

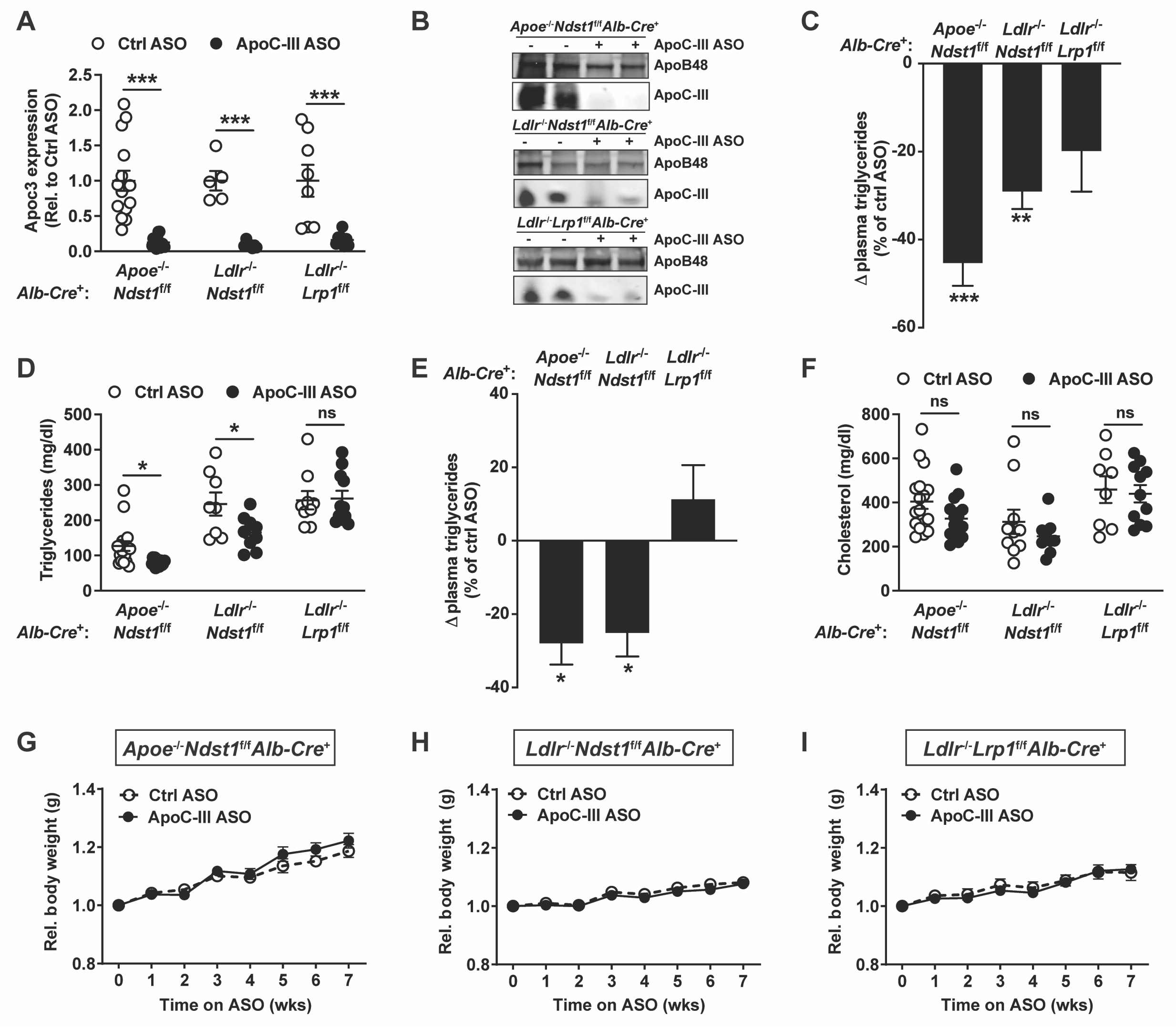

Supplemental Figure 1. Impact of lowering apoC-III on body weight and plasma lipid levels in mice fed a chow diet. Mice were treated with control ASO or apoC-III ASO (50 mg/kg/body weight) for 8 weeks. (**A**) Hepatic *Apoc3* gene expression in *Apoe*$^{-/-}$*Ndst1*$^{f/f}$*Alb-Cre*$^{+}$ (n = 18-19/group), *Ldlr*$^{-/-}$*Ndst1*$^{f/f}$*Alb-Cre*$^{+}$ (n = 13-16/group), and *Ldlr*$^{-/-}$*Lrp1*$^{f/f}$*Alb-Cre*$^{+}$ mice (n = 10-12/group) on either control or apoC-III ASO. Values are expressed relative to control ASO. (**B**) Pooled plasma samples (2 µl, n = 3/pool) were analyzed by Western blotting with antibodies against apoB and apoC-III. (**C**) Relative changes in plasma triglyceride levels after 8 weeks of

apoC-III ASO compared to control ASO. (**D**) Fasting plasma triglyceride levels and (**E**) relative changes in triglycerides compared to control ASO after 4 weeks of ASO treatment (n = 8-19). (**F**) Cholesterol levels after 4 weeks of treatment (n = 8-19). Relative body weight in (**G**) *Apoe*$^{-/-}$*Ndst1*$^{f/f}$*Alb-Cre*$^{+}$ (n = 18-19/group), (**H**) *Ldlr*$^{-/-}$*Ndst1*$^{f/f}$*Alb-Cre*$^{+}$ (n = 13-16/group), and (**I**) *Ldlr*$^{-/-}$*Lrp1*$^{f/f}$*Alb-Cre*$^{+}$ mice (n = 10-12/group). Data presented as mean ± SEM. Statistical differences between two groups were calculated using an unpaired two-tailed Student's t test. *$p<0.05$, ***$p<0.001$, ns: not significant.

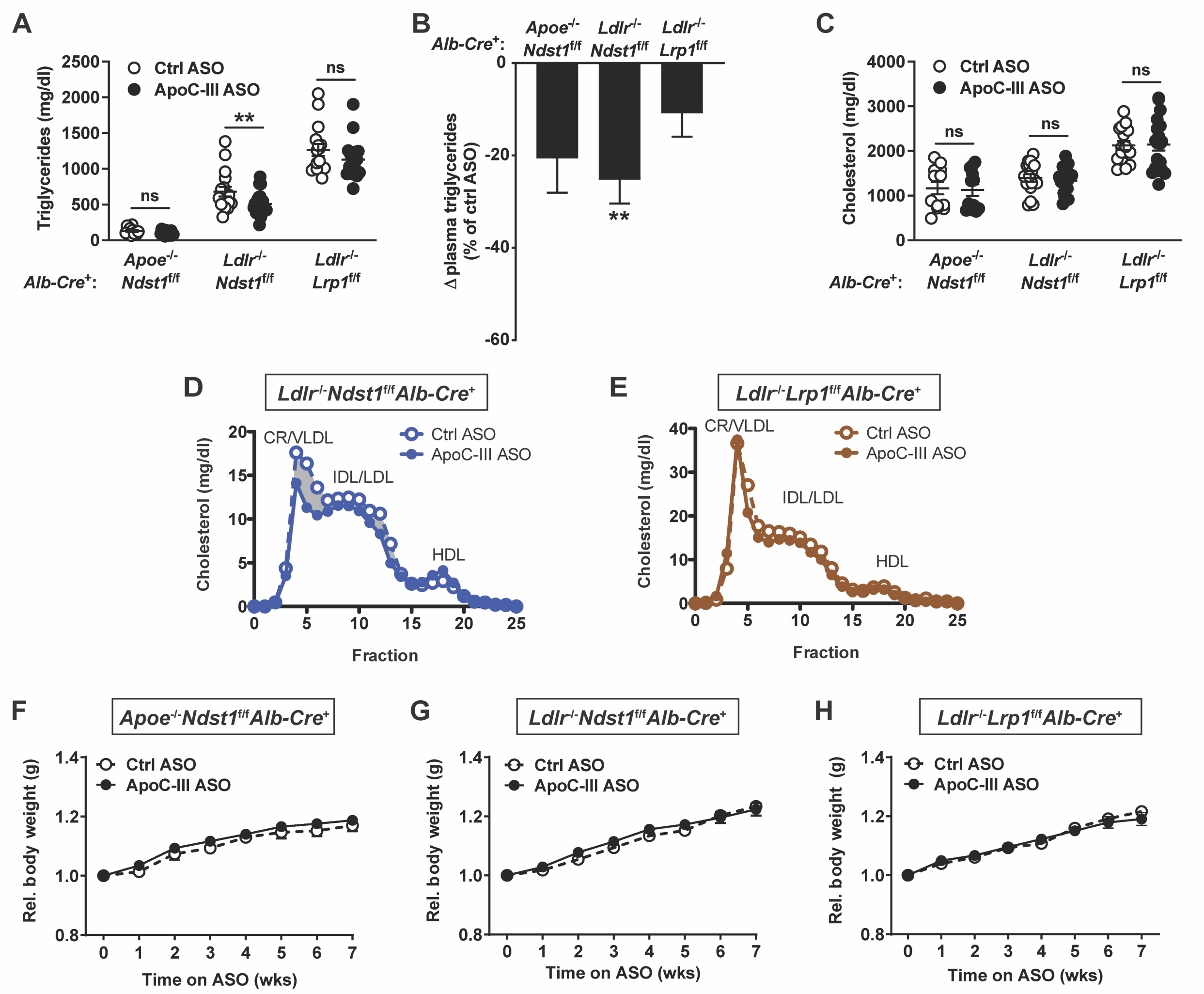

Supplemental Figure 2. Impact of lowering apoC-III on body weight and plasma lipid levels in mice fed a Western diet. (**A**) Fasting plasma triglycerides after 4 weeks of control ASO or apoC-III ASO in Western diet fed *Apoe*$^{-/-}$*Ndst1*$^{f/f}$*Alb-Cre*$^{+}$ (n = 11-13/group), *Ldlr*$^{-/-}$*Ndst1*$^{f/f}$*Alb-Cre*$^{+}$ (n = 18-22/group), and *Ldlr*$^{-/-}$*Lrp1*$^{f/f}$*Alb-Cre*$^{+}$ mice (n = 17-18/group). (**B**) Relative changes in plasma triglycerides compared to control ASO. (**C**) Fasting plasma cholesterol levels after 4 weeks of control ASO or apoC-III ASO. (**D**/**E**) Pooled plasma lipoproteins from fasted (**D**) *Ldlr*$^{-/-}$*Ndst1*$^{f/f}$*Alb-Cre*$^{+}$and (**E**) *Ldlr*$^{-/-}$*Lrp1*$^{f/f}$*Alb-Cre*$^{+}$ mice treated with ApoC-III ASO for 6 weeks were analyzed by FPLC (n = 3/pool). Relative body weight of (**F**) *Apoe*$^{-/-}$*Ndst1*$^{f/f}$*Alb-Cre*$^{+}$ (n = 11-13/group), (**G**) *Ldlr*$^{-/-}$*Ndst1*$^{f/f}$*Alb-Cre*$^{+}$ (n = 18-22/group), and

(**H**) *Ldlr*$^{-/-}$*Lrp1*$^{f/f}$*Alb-Cre*$^{+}$ mice (n = 17-18/group). Data presented as mean ± SEM. Statistical differences between two groups were calculated using an unpaired two-tailed Student's t test. ***p*<0.01, ns: not significant. Data for (**A**) and (**F**) for *Apoe*$^{-/-}$*Ndst1*$^{f/f}$*Alb-Cre*$^{+}$ mice are taken from (*17*) for comparison.

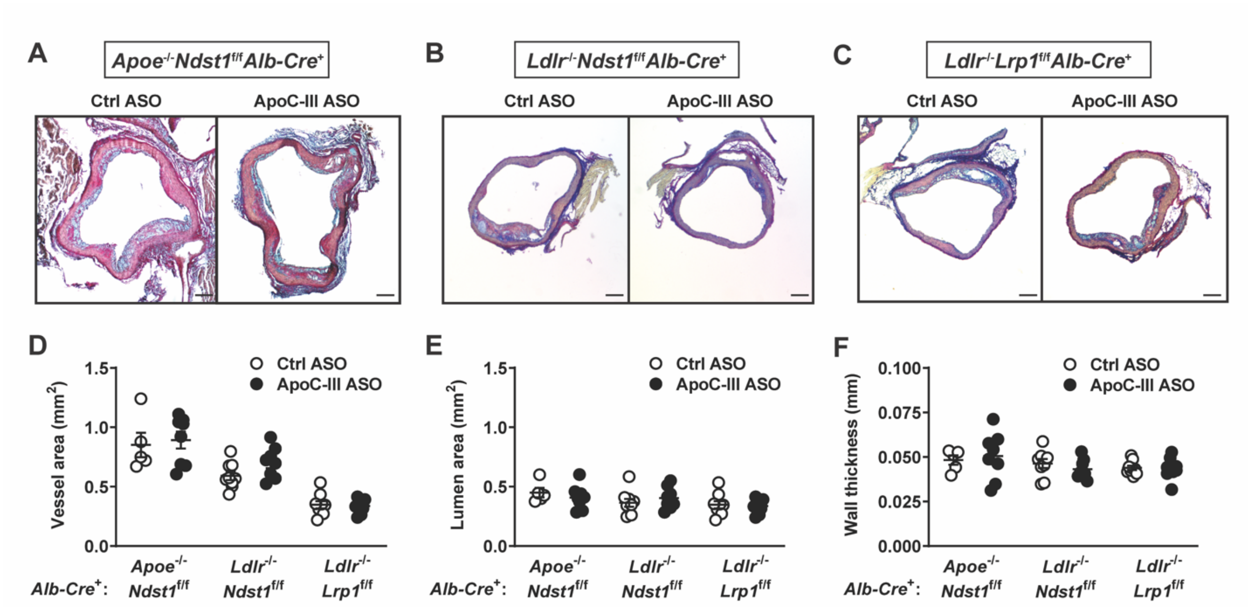

Supplemental Figure 3. Effects of ApoC-III ASO on parameters of the aortic vessel in mice fed a Western diet. Representative images of aortic root cross-sections stained with modified Verhoeff-van Gieson (800 µm) in (**A**) *Apoe*$^{-/-}$*Ndst1*$^{f/f}$*Alb-Cre*$^{+}$, (**B**) *Ldlr*$^{-/-}$*Ndst1*$^{f/f}$*Alb-Cre*$^{+}$, and (**C**) *Ldlr*$^{-/-}$*Lrp1*$^{f/f}$*Alb-Cre*$^{+}$ mice treated with control ASO or apoC-III ASO for 8 weeks. Quantification of the (**D**) aortic vessel area, (**E**) aortic lumen area, and (**F**) vessel wall thickness (n = 5-8/group). Data presented as mean ± SEM. Statistical differences in lesion size over distance were calculated using a two-way ANOVA with Bonferroni post hoc analysis. Statistical differences between two groups were calculated using an unpaired two-tailed Student's t test. Scale bars equal 100 µm.

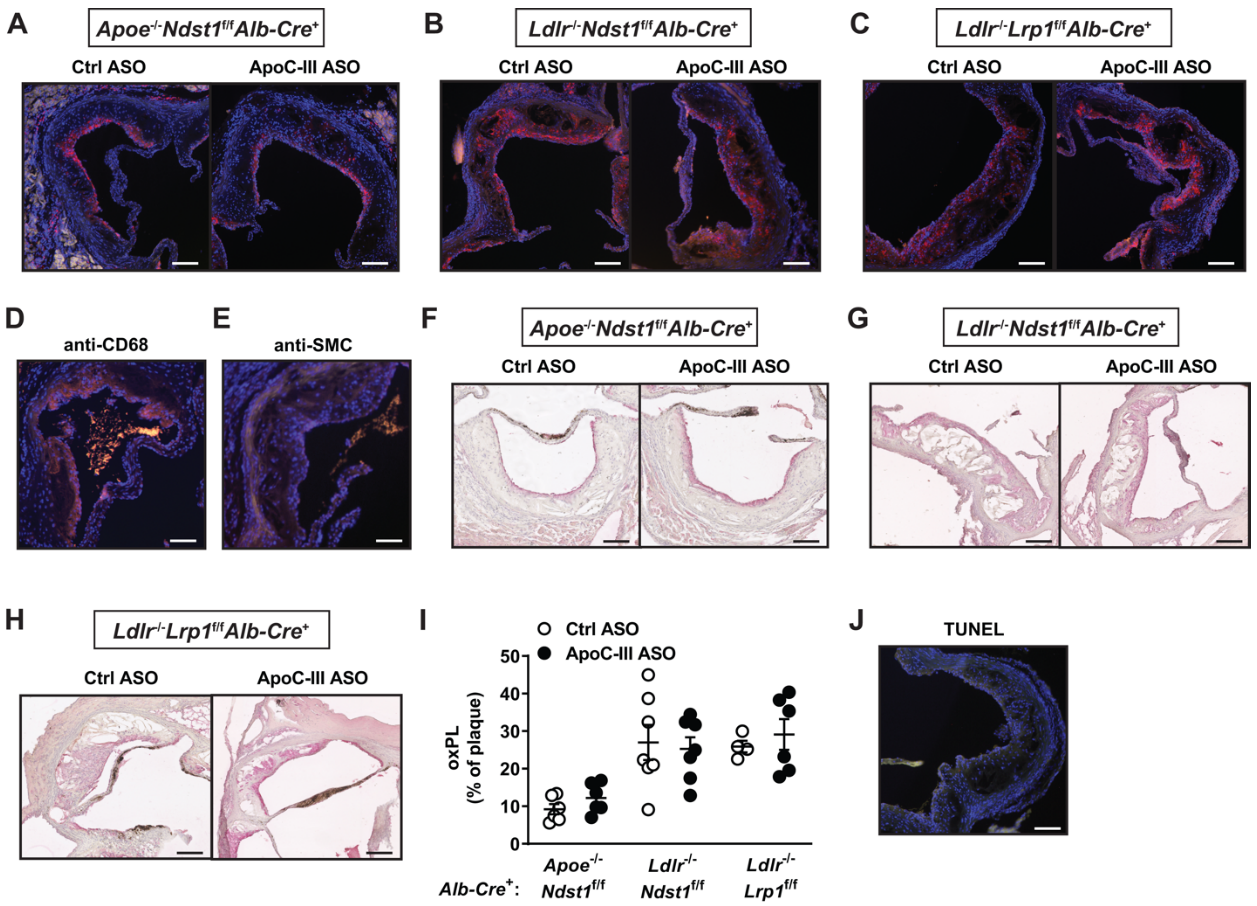

Supplemental Figure 4. Analysis of aortic root sections for CD68 and oxidized phospholipids in mice fed a Western diet. (**A-C**) Representative images of aortic root cross-sections (300 µm) of *Apoe*$^{-/-}$*Ndst1*$^{f/f}$*Alb-Cre*$^{+}$, *Ldlr*$^{-/-}$*Ndst1*$^{f/f}$*Alb-Cre*$^{+}$, and *Ldlr*$^{-/-}$*Lrp1*$^{f/f}$*Alb-Cre*$^{+}$ mice on Western diet after 8 weeks of control ASO or apoC-III ASO stained for CD68. (**D**) Negative control for macrophage CD68 stain. (**E**) Negative control for SMC stain. (**F-H**) Representative images of aortic root cross-sections (300 µm) stained for oxidized phospholipids. (**I**) Oxidized phospholipids were quantified using a biotinylated E06 antibody (n = 4-7/group). (**J**) Negative control for TUNEL stain. Scale bars equal 100 µm.

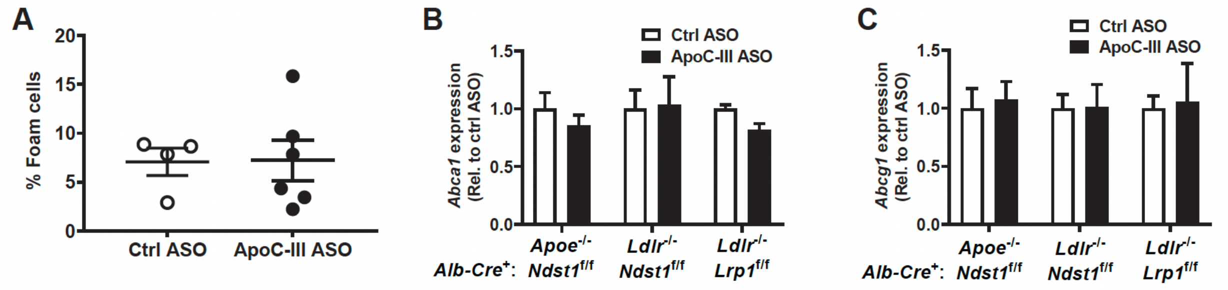

Supplemental Figure 5. Gene expression study of cholesterol efflux markers in aortic plaques of mice fed a Western Diet. *Ldlr*$^{-/-}$*Ndst1*$^{f/f}$*Alb-Cre*$^{+}$ mice on a Western diet were administered control ASO or apoC-III ASO for 8 weeks. (**A**) Peritoneal macrophages were isolated and foam cell formation was quantified using Oil Red O stain after 2 h of incubation at 37°C (n = 4-6/group). Gene expression of (**B**) *Abca1* and (**C**) *Abcg1* in the aortic plaque relative to control ASO in *Apoe*$^{-/-}$*Ndst1*$^{f/f}$*Alb-Cre*$^{+}$, *Ldlr*$^{-/-}$*Ndst1*$^{f/f}$*Alb-Cre*$^{+}$, and *Ldlr*$^{-/-}$*Lrp1*$^{f/f}$*Alb-Cre*$^{+}$ mice (n = 3-8/group). Data presented as mean ± SEM. Statistical differences between two groups were calculated using an unpaired two-tailed Student's t test.

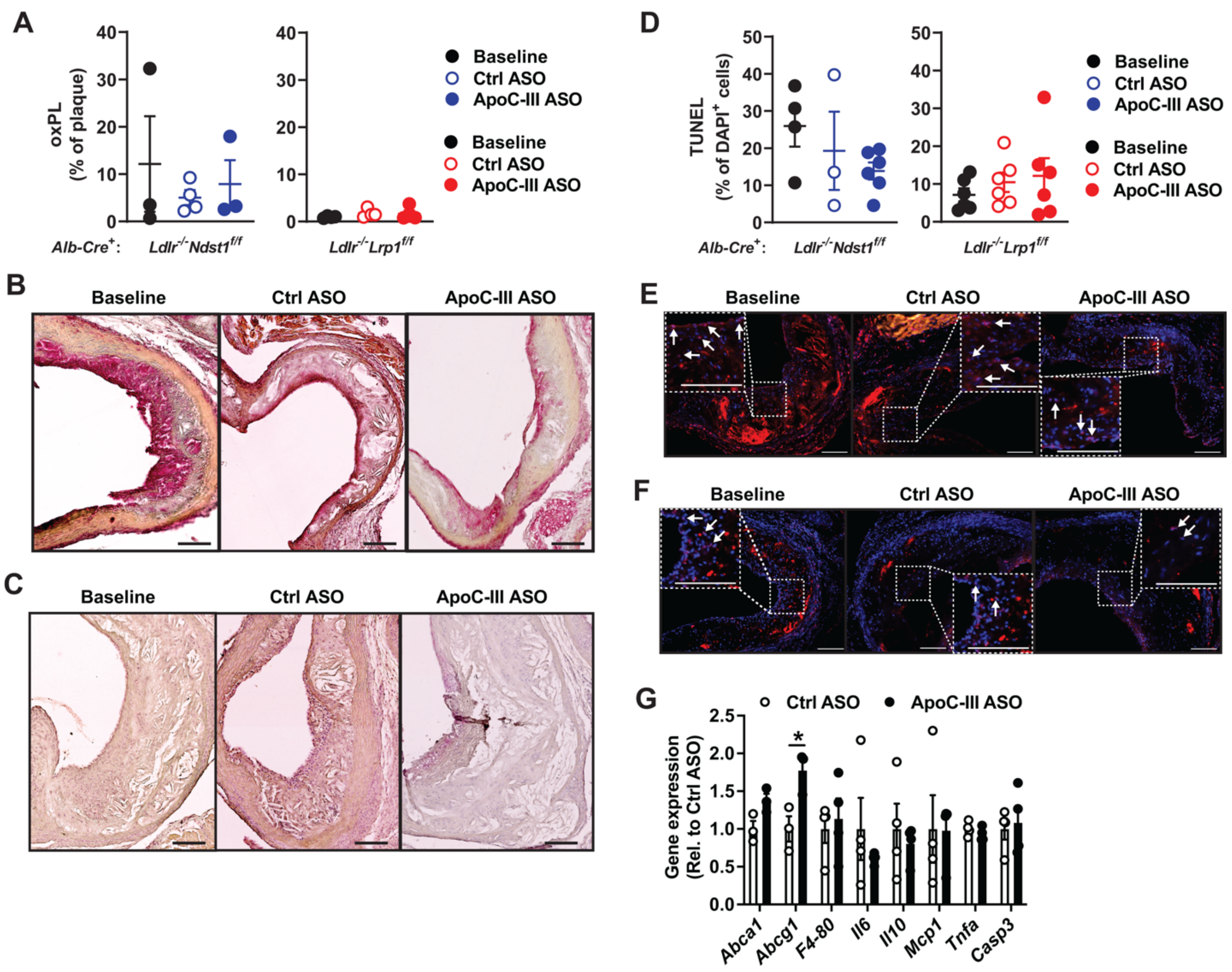

Supplemental Figure 6. Analysis of aortic root cross sections for oxidized phospholipids and TUNEL after diet intervention. Following diet intervention and administration of control ASO or apoC-III ASO, oxidized phospholipids and apoptotic cells were analyzed in aortic root cross-sections (700 µm). (**A**) Quantification of oxidized phospholipids using a biotinylated E06 antibody (n = 3-4/group). (**B**) Representative images for oxidized phospholipid stain in *Ldlr*$^{-/-}$ *Ndst1*$^{f/f}$*Alb-Cre*$^{+}$ and (**C**) *Ldlr*$^{-/-}$*Lrp1*$^{f/f}$*Alb-Cre*$^{+}$ mice. (**D**) Quantification of apoptotic cells using a terminal TUNEL stain and DAPI (n = 3-6/group). TUNEL positive cells were quantified relative to total DAPI positive cells. Example images are shown in (**E**-**F**). Arrows indicate examples of TUNEL positive cells. (**G**) Gene expression of cholesterol efflux genes (*Abca1*, *Abcg1*),

inflammatory markers (*F4-80*, *Mcp1*, *Il6*, *Il10*, *Tnfa*), and apoptosis (*Casp3*) in aortic lesions of *Ldlr*$^{-/-}$*Ndst1*$^{f/f}$*Alb-Cre*$^{+}$ mice were quantified relative to control ASO (n = 3-4/group). Data presented as mean ± SEM. Scale bar equals 100 µm. Statistical differences between three groups were calculated using a one-way ANOVA with Tukey post hoc analysis.

Supplement Table 1. Murine Antisense Oligonucleotide Sequences.

**Ionis ID**	**Name**	**Target Gene**	**Sequence**
ION 440726	ApoC-III ASO	*Apoc3*	5'-CCAGCTTTATTAGGGACAGC-3'
ION 141923	Control ASO	N.A. (scramble)	5’-CCTTCCCTGAAGGTTCCTCC-3'

Supplement Table 2: Real-Time PCR Primers.

**Gene**	**Forward Primer**	**Reverse Primer**
Abca1	5' CGTTTCCGGGAAGTGTCCTA 3'	5' GCTAGAGATGACAAGGAGGATGGA 3'
Abcg1	5' AGGTCTCAGCCTTCTAAAGTTCCTC 3'	5' TCTCTCGAAGTGAATGAAATTTATCG 3'
Apoc3	5’-TGCAGGGCTACATGGAACAA-3’	5’-TCGGACTCCTGCACGCTACTT-3’
Atf4	5’-GTTGGTCAGTGCCTCAGACA-3’	5’-CATTCGAAACAGAGCATCG-3’
Casp3	5’-AGATGGCTTGCCAGAAGATAC-3’	5’-CTGCAAAGGGACTGGATGAA-3’
Ddit3	5’-CTGCCTTTCACCTTGGAGAC-3’	5’-CGTTTCCTGGGGATGAGATA-3’
F4/80	5’-CTTTGGCTATGGGCTTCCAGTC-3’	5’-GCAAGGAGGACAGAGTTTATCGTG-3’
Il-6	5’-CCAGAGATACAAAGAAATGATGG-3’	5’-ACTCCAGAAGACCAGAGGAAAT-3’
Il-10	5’-TGAATTCCCTGGGTGAGAAG-3’	5’-TCACTCTTCACCTGCTCCACT-3’
Mcp1	5’-AGGTCCCTGTCATGCTTCTG-3’	5’-GCTGCTGGTGATCCTCTTGT-3’
Mfge8	5′-ATCTACTGCCTCTGCCCTGA-3′	5′-CCAGACATTTGGCATCATTG-3’
Tbp	5’-GAAGCTGCGGTACAATTCCAG-3’	5’-CCCCTTGTACCCTTCACCAAT-3’
Tnfα	5’-CCAGACCCTCACACTCAGATC-3’	5’-CACTTGGTGGTTTGCTACGAC-3’
